# Advances and Challenges in the Creation of Porous Metal Phosphonates

**DOI:** 10.3390/ma13235366

**Published:** 2020-11-26

**Authors:** Bharadwaj Mysore Ramesha, Vera Meynen

**Affiliations:** Laboratory of Adsorption and Catalysis (LADCA), Department of Chemistry, University of Antwerp, Universiteitsplein 1, 2610 Wilrijk, Belgium; vera.meynen@uantwerpen.be

**Keywords:** hybrid porous materials, layered metal phosphonates, phosphonate MOFs, supramolecular-templated porous metal phosphonates, separation and extraction, heterogeneous catalysis, proton conduction

## Abstract

In the expansive world of porous hybrid materials, a category of materials that has been rather less explored than others and is gaining attention in development is the porous metal phosphonates. They offer promising features towards applications which demand control over the inorganic–organic network and interface, which is critical for adsorption, catalysis and functional devices and technology. The need to establish a rationale for new synthesis approaches to make these materials in a controlled manner is by itself an important motivation for material chemists. In this review, we highlight the various synthetic strategies exploited, discussing various metal phosphonate systems and how they influence the properties of porous metal phosphonates. We discuss porous metal phosphonate systems based on transition metals with an emphasis on addressing challenges with tetravalent metals. Finally, this review provides a brief description of some key areas of application that are ideally suited for porous metal phosphonates.

## 1. Introduction

There has been a surge in the pursuit to create inorganic–organic hybrid materials in the past two decades, with the success of metal–organic frameworks (MOFs) and periodic mesoporous organosilicas (PMOs). Hybrid materials are interesting for material chemists and in application as the integration of both organic and inorganic functionalities allows to modify the matrix and interface properties of materials. The unique combination of inorganic and organic features creates particular properties and control with respect to the structure, physical properties and/or specific chemical reactivity and/or interactions. This has indeed resulted in the extensive and intensive creation of hybrid porous materials such as MOFs [1,2,3,4,5] and PMOs [6,7,8]. An interesting, more specific and less studied class of materials are porous metal phosphonates, built up from the interaction between metals and organophosphonic units. These hybrid porous metal phosphonate materials are particularly interesting where stable performance under sometimes harsh conditions and specific surface interactions are an important requirement for application, like the sorption of metal ions, heterogeneous catalysis, thin-film-based devices, chromatography or membranes technology.

**Figure 1:**
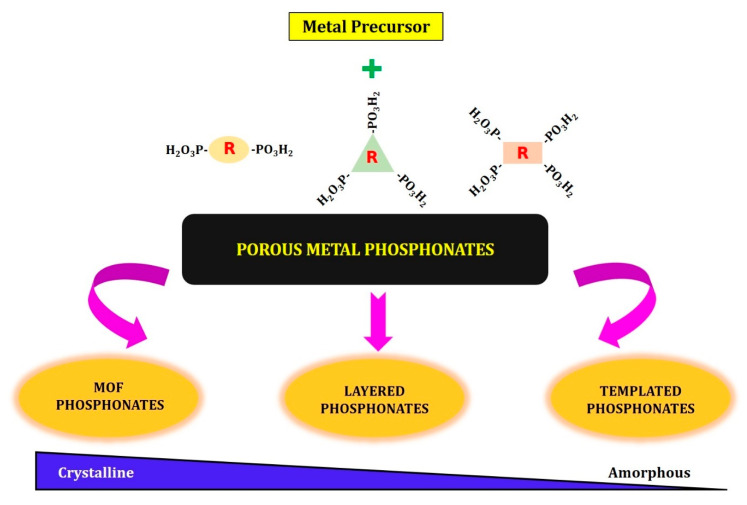
Scheme for classifying porous metal phosphonates based on structural/morphological properties.

We describe in this review the evolution of metal phosphonates over the past couple of decades from layered metal phosphonates to phosphonate-based MOFs and finally to templated (supramolecular) mesoporous metal phosphonates. All of these materials fall under the category of coordination polymeric networks and can be subdivided further based on their long-range order and/or pore morphology into: phosphonate MOFs, layered metal phosphonates and templated (supramolecular) metal phosphonates, as illustrated by Figure 1. This classification is thus only based on the final structure/texture of the porous coordination networks, which is primarily determined by the interaction of the metal precursors and phosphonic moieties in solution during synthesis, resulting in structures featuring a variety of coordination modes. We would like to point out that this is not a detailed review concerning each of these classes of materials, but rather an attempt to put together some of the similarities and differences when it comes to the choice of metal, metal valency and phosphonic linkers with respect to specific interesting morphologies of porous coordination networks that can be obtained. The critical aspects required to obtain particular metal phosphonate architectures are recognized by analyzing the reaction parameters and their consequences on the porous hybrid frameworks obtained. For a more detailed reading on the synthesis and/or applications for each class of metal phosphonates, we refer the readers to some of the interesting reviews [9,10,11,12,13,14,15,16,17].

Many of the interesting advances and developments in the field of porous metal phosphonates hybrids have taken place in the last couple of decades. A substantial part of the progress in porous metal phosphonates has been built on the knowledge stemming from the pioneering works from Clearfield et al. and Alberti et al. on layered metal phosphates/phosphonates [9,10,18,19,20]. We describe an overview of the different synthesis methods undertaken for various transition metals and then funnel down towards the metal (IV) phosphonate system. The focus of this review is addressing the challenges posed for creating porous metal (IV) phosphonate networks. We restrict ourselves to materials synthesized purely from transition metal precursors and organic precursors containing only phosphonate moieties. Therefore, mixed ligand systems such as the metal carboxyphosphonates are not a part of this review [21]. This review consists of two parts, where we first summarize on the synthesis methods employed for obtaining porous metal phosphonates and subsequently provide an overview of the potential benefit of porous metal phosphonates in some key areas of application.

## 2. Synthesis of Porous Metal Phosphonates

In this section, we will discuss some of the metal phosphonate networks that have been created in the past couple of decades leading up to the metal (IV) phosphonates and the challenges faced in obtaining these metal phosphonate networks. The synthetic conditions of the porous metal phosphonates together with the choice of precursor(s) and type of organophosphonic acid define the type of material that is ultimately desired, which we discuss in this review as a part of the three different structural/morphology classes (Figure 1):

(1) Layered metal phosphonates;
(2) Phosphonate–metal organic frameworks;
(3) Supramolecular templated porous metal phosphonates.

The synthetic details for each class of materials, namely layered metal phosphonates, phosphonate-based MOFs and templated porous metal phosphonates, have been summarized in the Supplementary Information (Tables S1, S2 and S3, respectively).

### 2.1. Layered Metal Phosphonates

Layered metal phosphonates were synthesized by the direct precipitation between metal precursors and organophosphonic acids in a similar way as known for metal phosphates [9,10]. The resulting materials were found to be analogous in structure to layered metal phosphates, but with the interlayer distance depending on the organic linker group [22,23]. As in the literature “metal organophosphonates” are often described as “metal phosphonates”, we will from here on leave out “organo” even though these materials are hybrid materials synthesized from metal and organophosphonic precursors. 

In this section, we will discuss the syntheses of metal phosphonates of divalent/trivalent metals and end by discussing tetravalent metal phosphonates. The key difference between those two relates to the solubility of metal ions in solutions. Transition metals strongly interact with phosphonic acid precursors and form an insoluble coordination polymer. However, a prerequisite to obtain a crystalline structure is a controlled, ordered buildup of molecular precursors into a hybrid network, requiring the metal moieties to be solubilized in sufficient quantity, allowing homogeneous interactions with the phosphonic precursors. In the case of metals that are divalent/trivalent, this is achieved by lowering the pH of the reaction medium, and thus synthesizing in an acidic condition. However, with tetravalent metal precursors, the formation of a metal phosphonate coordination network is kinetically too fast, resulting in the precipitation of the mixtures in an uncontrolled way, even in high acidic conditions and therefore hampering controlled structural properties [24]. One of the ways to overcome this has been by the introduction of HF as a solubilizing/mineralizing agent, whereby F^−^ ions form a complex with metal ions, causing a slow release of metal ions into the solution available for interaction with the phosphonic precursor upon heating under pressure [20].

With respect to divalent metals, zinc (II) diphosphonates formed by phenylene and bisphenylene phosphonic linkers resulted in a structure that was found to be dependent on the length of the aryl linker. In the case of the monophenylene linker, the phenylene phosphonate groups functioned as pillars between the octahedral zinc oxide layers. However, in the presence of biphenylene as a linker, the inorganic unit formed chains rather than a layered structure with the zinc being coordinated tetrahedrally. The same phenomena were observed with copper (II) diphosphonates with phenylene and bisphenylene linkers [25,26]. Moreover, when using non-aromatic linkers, it was reported that the structure of the materials was sensitive to the length of the linker/spacer in copper (II) and zinc (II) diphosphonates made from ethylene diphosphonic acid or propylene diphosphonic acid. The presence of an extra methylene unit leads to a change in the coordination of copper and zinc from an octahedral layered into a tetrahedral chain structure. Hence, the length of linker moiety (alkyl or aryl) has pronounced consequences on the coordination of the linkers to the metal-oxo centers and thus dictates the final structure of the material. These key structural features in layered materials were determined by single-crystal X-ray diffraction and was therefore important to understand the details of the coordination geometry with different linkers [27]. 

Different types of precursors in combination with variations in synthesis conditions were found to influence the structural properties of layered metal phosphonates. The use of diphosphonic acids containing an amine group allows to tune the coordination with the metal, based on the reaction conditions and the choice of metal precursor, which influences the formation of the network. Zinc (II) and manganese (II) diphosphonates were synthesized with N-ethylimino bis(methylene phosphonic acid), yielding a 2D-layered structure with Mn (II) and a 3D network when Zn (II) was the metal source. The degree of deprotonation of the phosphonic linker and the affinity of the metal for the amine group was found to dictate the way the hybrid structure evolved during the synthesis [28]. Another example illustrating the importance of reaction conditions was demonstrated by the reaction of cobalt (II) and octylene diphosphonic acid. The room temperature synthesis of the precursors in HF resulted in a clathrate structure made of 1D chains featuring a cobalt atom and undissociated diphosphonic acid in a polymeric network held together by hydrogen bonds. However, when the same system was subjected to a hydrothermal treatment (in the absence of HF), layered cobalt (II) diphosphonate with octyl chains in the inter-layer were formed. Therefore, depending on the synthesis conditions and presence or absence of HF, different structures were formed. The degree of protonation plays an important role in the hydrogen bonded networks in such systems [29].

Due to the closed packing of the phosphonic linkers as pillars, the metal phosphonates did not possess any porosity. Nevertheless, porous systems are known to have higher surface areas and provide better interfacial properties that are crucial for applications such as catalysis, gas adsorption, separation/extraction and others. Therefore, porosity is often a highly desired property and we therefore describe how porosity can be introduced in these layered metal phosphonate systems. These methods involved the use of additives or mixing solvent systems as will be discussed in the following paragraphs. 

A strategy to impart porosity in the layered materials was by spacing out the closely packed inter-layer spacers through the use of organic linkers with over-crowded diphosphonic acids. This created inorganic layers (e.g., Cd (II) and Ni (II) oxides) with anchored organic groups pointing inwards between the layers but without close packing. Nevertheless, these materials were still not possessing any (accessible) porosity [30]. In another approach to achieve porosity in layered metal phosphonates, zinc (II) precursor and phenylene and biphenylene diphosphonic acid were reacted in the presence of varying amounts of phosphoric acid in the synthesis mixture. It was shown that indeed, a three-component system with a suitable phosphonate/phosphate (1:4 and 1:7) ratio resulted in a material that had inter-particle slit-shaped porosity in the mesopore region (~6.6 nm). It was noted that an equal phosphonate/phosphate ratio resulted in a material that was purely zinc(II) phosphonate, while higher phosphate incorporation led to the formation of zinc phosphate. However, with the right amount of phosphoric acid, phosphate moieties were incorporated between the phenylene linkers at random places without altering the crystal structure, resulting in a mesoporous texture [31]. 

The use of metals like Ca, Zn, or Mn (valency < 3) with phosphonic acids usually leads to materials that exhibit a layered morphology [32]. Therefore, the same strategy as in divalent metals was employed to impart porosity in zirconium (IV)-based metal phosphonates. The Zr (IV) precursor was reacted with 4,4′-biphenyldiphosphonic acid in the presence of phosphoric acid that would act as a spacer between the aryl linkers [20]. A mechanism of a house-of-cards arrangement was proposed, creating porosity between the layered particles of zirconium bisphosphonate–phosphite materials. The inter-particle mesoporosity was achieved by balancing the amount of phosphoric acid/diphosphonic acid ratio [33]. However, as tetravalent metals have the problem of insolubility (e.g., zirconium and titanium metals) caused by the rapid hydrolysis and condensation of metal (IV) precursors resulting in rapid precipitation when they have reacted with phosphonic acid species, the crystallinity of these layered metal phosphonates seems to be improved only when the synthesis was performed in the presence of HF as the solubilizer/mineralizer [20]. The porosity in these materials was mainly dictated by the packing density of the organic linker between the inorganic metal oxide layers, acting as spacers, and on the presence of ingredients such as solvent/additives in the synthesis recipe [9,10]. Attempts to make narrow pore size lamellar metal phosphonates were achieved by Alberti et al. by replacing around 30% of the butane and benzene diphosphonic acid moieties by a phosphite moiety in between the zirconium oxide layer. This way, mesoporosity was observed with pore sizes around 2–4 nm, depending on the amount of phosphite incorporated [18]. Similarly, complex pillaring moieties were incorporated, such as the 3,3′,5,5′—tetramethylbiphenyldiphosphonic acid that has bulky ends with narrow biphenyl units [19]. This material possessed a high surface area of 375 m^2^/g with inter-layer microporosity (with a pore size of ~0.5 nm) [34].

**Figure 2:**
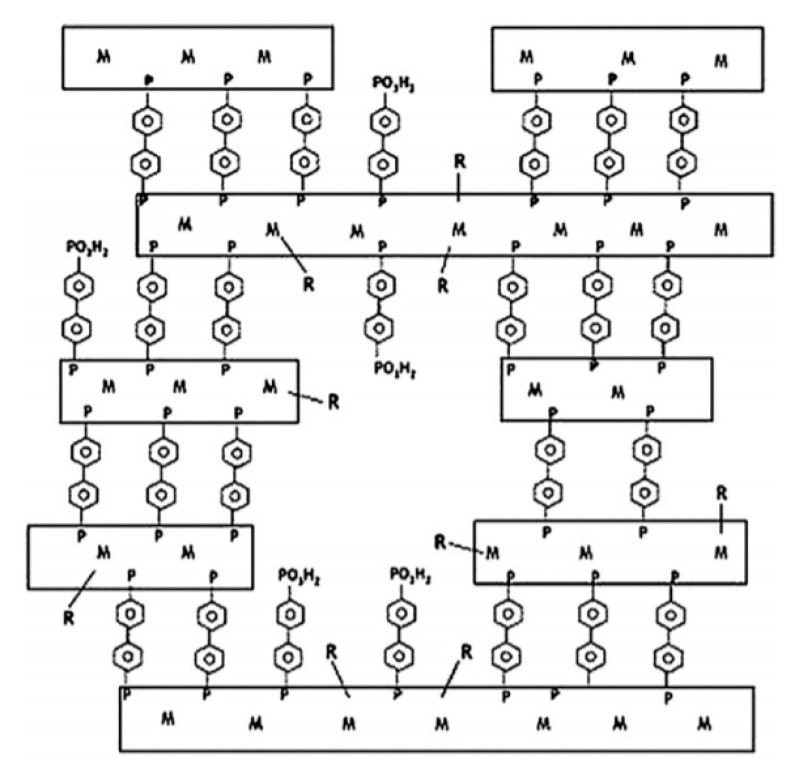
Schematic drawing of how micropores are formed in the metal biphenylene bis(phosphonates). The horizontal rectangles represent the inorganic layers (M = Zr or Sn) which are cross-linked by the organic moieties. R stands for F^−^, if HF is used in the preparation, and the surfaces are capped by M because an excess of metal is used in the syntheses. Reprinted with permission from [39]. Copyright (2009), Royal Society of Chemistry.

Tin (IV) phosphonates were prepared that consist of small nanoparticles with microporosity due to inter-particle voids that created tunnels in dimensions of 1.1–2.4 nm, depending on the size of the organic group of the phosphonate [35]. A similar attempt to synthesize porous layered material with tin (II) was unsuccessful even with the inclusion of mixed phosphonic ligands [36]. In contrast, a mixed phosphonic acid precursor of phenyl phosphonic acid and methyl phosphonic acid with tin (IV), resulted in mixed micro/mesoporosity. The stacking of tiny nanoparticles of tin (IV) phosphonates that was arranged in a house-of-cards fashion contributed to the mixed porosity. The porosity was found to be dependent on the ratio of phenyl phosphonic/methyl phosphonic acid amount and on the presence or absence of HF. In the absence of HF, the materials were mesoporous and in the presence of HF, the materials showed microporosity [37]. In another study, layered tin (IV) phosphonates that were synthesized from diphosphonic acids had completely different structures in comparison with tin (IV) phosphonates from monophosphonic acids. Tin (IV) phosphonates with diphosphonic acids presented larger particles with sizes ~20 µm, while tin (IV) phosphonates with monophosphonic acids were ~2 µm in size. Thus, the nature of the phosphonic acids is crucial to particle growth in forming tin (IV) phosphonates. Along with the mono- or di-functionality of the phosphonic acid, the metal source plays an important role. With zirconium (IV), diphosphonic acids were inserted between the inorganic layers as pillars, whereas in the case of tin (IV) they were found to be incorporated in between inorganic layer as pillars as well as inserted as a part of the inorganic–organic chains. Because of this dual role, when tin (IV) was the metal, not all diphosphonic moieties were present as pillars and therefore voids were present in the layers which resulted in porosity in the final material [38]. In another study, the growth of porous layered tin (IV) phosphonates was explained by random cross-linking of layered species providing inter-layer porosity. A possible hypothesis for the underlying mechanism that caused the random stacking instead of layer growth was the presence of stacking defects by the combination of diphosphonate and monophosphonate ligands or the presence of protonated phosphonic acids [39]. Based on these results, a generalized growth model for metal (IV)diphosphonate coordination networks was proposed, resulting from rapid precipitation of unevenly grown capped plates, leading to formation of structure as illustrated by Figure 2 [39,40].

Apart from other factors such as the choice of the phosphonate ligand and the presence or absence of HF, also the type of solvent used plays a role in obtaining porous metal phosphonates. The obtained porosity was found to be dependent on the solvent system employed. Zr (IV) precursors are soluble in DMSO but poorly soluble in an alcohol/water medium, and the vice versa for Sn (IV) precursors. Based on the choice of alcohol (EtOH, PrOH, or BuOH) the porosity could be tuned towards microporous Sn (IV) bisphosphonates. However, for zirconium (IV) bisphosphonates, it was only possible when DMSO was used as the solvent [41]. 

### 2.2. MOFs–Phosphonates

Metal-organic frameworks are one of the prominent classes of coordination polymeric networks that are built-up from metal nodes and organo-functional units resulting in porous structures with very high surface areas. Here, we focus on a specific type of MOFs that consists of metallic units with phosphonate linkers. Frameworks based on metal phosphonates are rather an emerging class of MOFs when compared to the well established carboxylates, whereas the surge in carboxylate MOFs has been on a continuous rise ever since their inception in the late 1990s. The successful synthesis of frameworks based on metal phosphonates (albeit small in number) has only been possible in this decade. This can largely be attributed to the stronger coordination of the phosphonate moiety towards metals than the carboxylate counterparts [11]. Therefore, hindering the orderly growth of a porous metal phosphonate framework. 

We begin this section by discussing some of the phosphonate frameworks, similar to layered metal phosphonates: divalent/trivalent metal phosphonate frameworks that have led to the development of tetravalent metal phosphonate frameworks. One can expect that similar to layered structures, the problem of solubility is very apparent and impedes creating porous architectures. One finds in the literature that MOFs that have been deemed as the ‘ultra-stable’ systems are mostly composed of Ti (IV) and Zr (IV) nodes [24]. Thus, it is crucial in studying how these frameworks could be obtained with phosphonic linkers. In this section, we build on the knowledge of layered metal phosphonates and describe how their lamellar motif can be altered, going towards open porous architectures.

A 3D framework synthesized from zinc and 1,4-dihydroxy-2,5-benzenediphosphonic acid in DMF resulted in the formation of 1D chains of zinc tetrahedra and RPO_3_^2−^ with a surface area of 209 m^2^/g (microporous with 1 nm × 1 nm pores), once DMF was removed from the framework. The key component of the phosphonic linker, the dihydroxy group, prevented the formation of a dense layered motif and thus resulted in an open framework [42]. The reaction of ethylenediaminetetrakis (methylenephosphonic acid) with lead (II) and zinc (II) also resulted in two different types of 3D open frameworks that were microporous. This phosphonic ligand containing eight phosphonic acid sites provided myriad coordination modes that were dependent on the choice of metal and the pH of the reaction medium. The framework based on lead was made up of helical chains of square-pyramidal lead and RPO_3_ phosphonate units. However, in the case of zinc, the amino group was not involved in binding with the metal and resulted in a square-wave type arrangement of zinc and the phosphonate layer. Both these frameworks possessed open channels in the micropore region (0.68 nm × 1 nm) [43]. 

**Figure 3:**
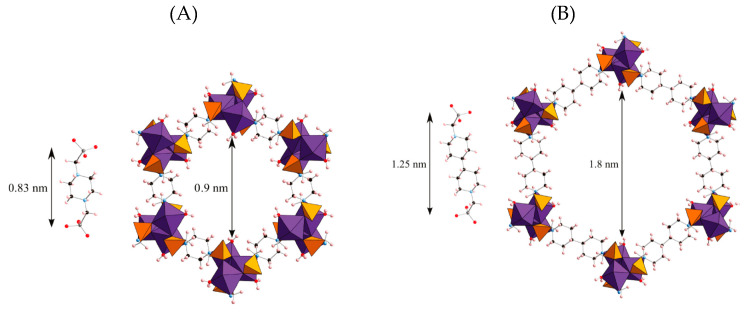
Frameworks of STA-12 (**A**) and STA-16 (**B**) viewed along the channel axis. Color code: cobalt: purple, phosphorus: orange, oxygen: red, nitrogen: blue, carbon: black, hydrogen: pink. Reprinted with permission from [51]. Copyright (2011), American Chemical Society.

**Figure 4:**
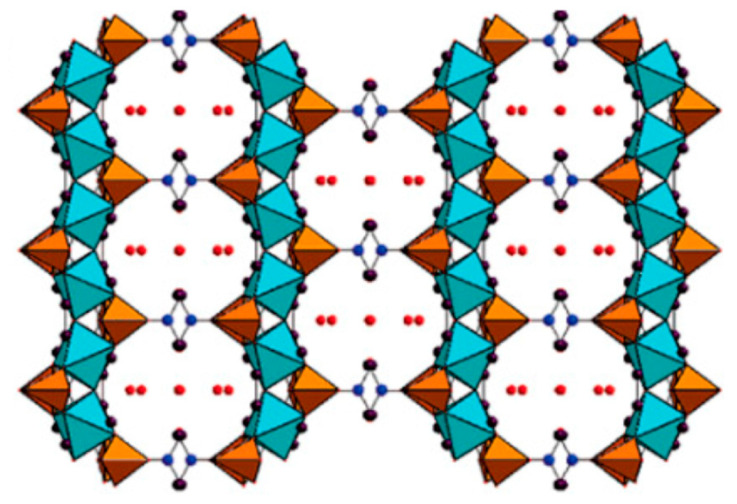
Structure of MIL-91(Ti) viewed along the c axis. Color code: titanium octahedra: cyan, phosphorus tetrahedra: orange, oxygen: red, nitrogen: blue, and carbon atoms: purple. Reprinted with permission from [60]. Copyright (2006), American Chemical Society.

**Figure 5:**
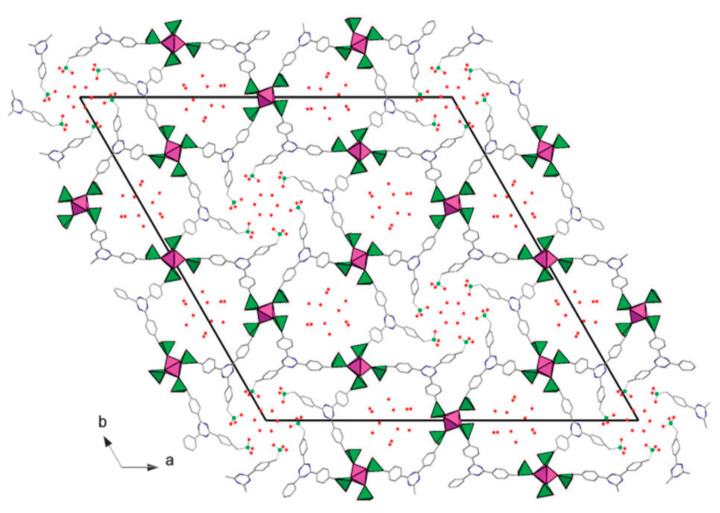
The crystal structure of UPG-1 viewed along the c axis. Color code: zirconium octahedra: purple, coordinating PO_3_C tetrahedra: green. Reprinted with permission from [63]. Copyright (2014), The Royal Society of Chemistry.

Based on the reaction conditions and the choice of metal and phosphonic precursors, a wide variation in coordination modes between the metal units to the phosphonic acid units can be obtained (see for example Figure 3 and Figure 4 versus Figure 5). When additives are added, novel coordination modes can be formed. For example, a framework with 1D pores was synthesized from benzene diphosphonic acid and copper (II) in the presence of amino triazole as an additive. This framework features a pillared structure with apparent pores of ~0.45 nm. It could only be formed due to the amino group of the triazole coordinating the copper atoms and producing 1D columns of 6-coordinated copper. The absence of the amino group of the triazole lead to the classical pillared copper benzene diphosphonate structure with no interlayer porosity [44]. In another study, the reaction of lead (II) with organo-piperazine as a diphosphonic ligand resulted in an initial 2D layered structure. This 2D structure was then transformed into a 3D framework via supramolecular assembly during the synthesis, due to the weak interaction of Pb–O(N) contacts. The formation of the supramolecular assembly leading to the obtained framework was attributed to hydrogen bonds, van der Waals and some electrostatic interactions [45]. 

Modifying the phosphonate ligands into R[PO(OH)(OR)]_2_, by derivatizing one of the acidic P–OH groups into a P–OR group, mimics a carboxylate ligand. Thus, this type of precursors is expected to allow the formation of secondary building units (SBU) that are well known from carboxylate MOFs. This concept was first employed with zinc(1,4-benzenediphosphonate-bis(monoethyl ester)), to create a pillared structure with the ester moiety pointing inwards between the layers and with permanent porosity (aperture ~0.6 nm) due to the Van der Waal forces between the alkyl chains in the interlayer [46]. The same phosphonic linker was later used to synthesize frameworks based on copper, and in this case, the linker possessed both methyl and ethyl ester groups. The resulting structure of the copper phosphonate framework with the ethyl ester was similar to the previous example with zinc. The use of these phosphonate linkers, mimicking a carboxylate linker, has led to the creation of porous frameworks built up via SBUs. These phosphonate MOFs also have advantages in application as they, for example, showed good affinity for CO_2_ with the high isosteric heat of adsorption of 45 kJ/mol similar to carboxylate MOFs such as HKUST-1 (35 kJ/mol), MIL-101 (44 kJ/mol), MIL-100 (63 kJ/mol) and Mg-MOF-74 (47 kJ/mol) [47]. 

Larger pore metal phosphonate framework synthesized from nickel (II) and copper (II) with N,N’-piperazinebis(methylenephosphonic)acid as the linker have been prepared, consisting of a hexagonal array of channels with a pore size of 1 nm that was filled with physisorbed water. However, upon dehydration, the framework distorted. When using a 2-methylated derivative of the same phosphonic linker, this structural distortion could be prevented and the framework retained its structure [48]. Reticular syntheses are described as the process of assembling rigid molecular building blocks into ordered networks, held by strong bonds [1]. Thus, to attain larger pores, isoreticular methods have been employed to synthesize large pore metal phosphonate MOFs in materials such as STA-12 with an isoreticular pore size of 0.9 nm. They were produced by the reaction of N,N′-piperazine bis(methylenephosphonic acid) with divalent metals (Mn, Fe, Co and Ni) [49,50]. To obtain larger pores, pushing towards the mesopore region, linkers such as the N,N′-4,4′-bipiperazine bis(methylenephosphonic acid) were reacted with cobalt to obtain 3D frameworks that have pores of 1.8 nm (STA-16). This 3D framework was composed of CoON_5_ octahedra as a spiral, which resulted in hexagonal pores with a 1.8 nm pore opening as shown in Figure 3 [51].

Phosphonate MOFs stable in water were synthesized from barium and octaethyl pyrene-1,3,6,8-tetraphosphonate resulting in a permanently porous framework denoted CALF-25. It is a three-dimensional framework made up of 1D inorganic–organic chains of barium phosphonate that are cross-linked by pyrene ligands. The pores of this framework consist of 1D rectangular openings of 0.46 nm × 0.39 nm containing hydrophobic ethyl chains [52]. Alternatively, a framework built up from tin and a triphosphonate ligand resulted in an amorphous, highly robust, and porous system having a surface area of 500 m^2^/g and pore width of 0.85 nm (CALF-28) [53,54]. In another study, the use of flexible tritopic phosphonate linkers provided the platform to synthesize materials of 2D and 3D frameworks or even 0D dimers with various metals (Cu, Ni, and Mn). Benzene-1,3,5-triyl- tris(methylene)triphosphonic-acid (BttP) or 2,4,6-trimethyl-BttP were used as the flexible linkers with a rigid aromatic core. It was shown that flexible tritopic phosphonate linkers allow a tunable structure of the framework, based on the choice of metal, pH, and the synthesis conditions, to end up with either a 2D or 3D architecture. The flexibility in the triphosphonate moiety allowed the linkers to adopt various coordination environments in bonding to different metals, influencing the conformation of the triphosphonate moiety and the resulting structure of the metal phosphonate framework [55]. 

Finally, phosphonate MOFs made from metal (IV) precursors are summarized in the following paragraphs. Phosphonate-based MOFs based on metal (IV) have been extremely difficult to prepare since they usually form a dense, amorphous layered structure. MIL-22 is a titanium (IV)-based 3D layered framework, wherein hydrous titanium oxide was reacted hydrothermally with ethylene diphosphonic acid. The inorganic–organic chain built up by Ti–O–Ti and Ti–O–P bonds, containing narrow channels that are filled with water in the interlayer region, provide minimal to no porosity. However, an important property was the excellent thermal stability of MIL-22 compared to other open-frameworks [56]. These MIL-22 structures were derived from the metal phosphonate frameworks synthesized with vanadium and alkyl diphosphonic acids [57,58,59]. However, by employing N,N′-piperazine bismethyl phosphonic acid, the frameworks MIL-91 (Ti (IV) and Al (III)) were synthesized. The MIL-91-Ti framework consists of MO_6_ octahedra and bisphosphonate groups having a surface area of ~500 m^2^/g, pore volume of 0.19 cm^3^/g and pores of size 0.35 nm × 0.40 nm. This was the first successful open phosphonate framework made from titanium(IV) as the metal node, shown in Figure 4 [60].

In another study using the same linker, piperazine-N,N′-bis(methylenephosphonic acid) and zirconium(IV), two 3D frameworks, were synthesized. The structure of these frameworks was found to be dependent on the pH of the reaction medium. At higher pH, a 3D framework was created by zirconium octahedra bonded to RPO_3_, forming composite building units (CBU), linking the inorganic–organic chains through the piperazine units resulting in the formation of a zirconium phosphonate framework. At a lower pH, the formation of the classic pillared-layered structure was observed [61]. Alternatively, a robust honeycomb-layered framework of zirconium triphosphonate was synthesized with 1,3,5-tris(4-phosphonophenyl)benzene as the linker by direct precipitation in the presence of HF. Each layer consisted of zirconia octahedra linked to RPO_3_ tetrahedra that acted as the SBU to form the honeycomb-layered framework. Even though the framework had good hydrolytic stability, it lacked porosity due to the capping of the layers [62]. Therefore, in order to induce porosity, the linker was replaced by 2,4,6-tris(4-phosphonomethyl)-phenyl 1,3,5-triazine (ttbmp), which is a flexible phosphonate ligand compared to the previous rigid linker. The resulting open framework UPG-1 was composed of 1D polymeric chains of zirconia octahedra linked by ttbmp, creating two kinds of channels: a 1 nm channel lined with free P–O groups and a smaller channel of 0.5 nm lined by uncoordinated PO_3_C groups as shown in Figure 5 [63]. In a similar manner, an aluminum phosphonate framework with a pore size of 1.24 nm was obtained [64].

The importance of hydrogen bonding in metal phosphonate structures has recently been clearly illustrated by Taddei et al. [65]. In this work, the conformational effect of phosphonic linkers on the evolution of the structure of the framework was shown. Two distinct structures were obtained as a result of the position of the (–CH_2_–PO_3_H_2_) moiety in either para- or meta- location on the novel triazine linker, which influenced the hydrogen bonding and therefore, resulted in a new zirconium phosphonate framework, UPG-2 with unique proton conducting behavior. 

### 2.3. Templated Porous Metal Phosphonates

The final part of the synthesis section is committed to porous metal phosphonates wherein the porous texture is created by the incorporation of templates (supramolecular) as structure-directing agents (SDAs). These materials take inspiration from the PMO hybrid system but follow a different approach as the metal oxide and organophosphonate precursors are incorporated into a hybrid network by a heterocondensation step. The protocol to make these mesoporous metal phosphonates is mostly based on the methodologies of porous metal oxide systems [66]. In general, porous metal oxides are obtained by the formation of metal–oxo oligomers around SDAs in solution or alternatively an evaporation-induced oligomer assembly with SDA triggered by the evaporation of the solvent, denoted as the evaporation-induced self-assembly method (EISA). Finally, a thermal step is applied to crystallize the metal oxide and to remove SDA molecules [67]. Alternatively, the SDA can be removed by extraction with/without a thermal step to enhance the condensation/crystallization [66,67]. Other synthesis methods for this class of materials have been adapted from the sol–gel approaches used to synthesize metal oxides. The sol–gel based materials form in most cases an amorphous metal oxide network that requires a final calcination step for crystallization [66,67]. However, in the case of hybrid materials possessing organic moieties embedded in the inorganic–organic network, calcination often cannot be applied for crystallization when the temperature required exceeds the thermal stability of the organic functional group. In these cases, extraction with or without a mild thermal treatment step are required. 

Templated metal phosphonate networks are often characterized with a texture possessing no long-range order (amorphous). One of the earliest mesoporous metal oxides in the presence of an inorganic phosphorus precursor was created by reaction of titanium isopropoxide with phosphoric acid in the presence of octadecyltrimethyl ammonium chloride/bromide as the SDA. This material consisted of an amorphous network of Ti–O–P with a surface area of 700 m^2^/g and pore sizes of 2.7–3.3 nm [68]. There have been many mesoporous metal phosphates made by the use of phosphoric acid without the presence of any organic functional groups [69,70,71]. Although valuable materials, these types of materials are not the subject of this review, and focus will be devoted to those materials made from a combination of metal and organophosphonic precursors.

**Figure 6:**
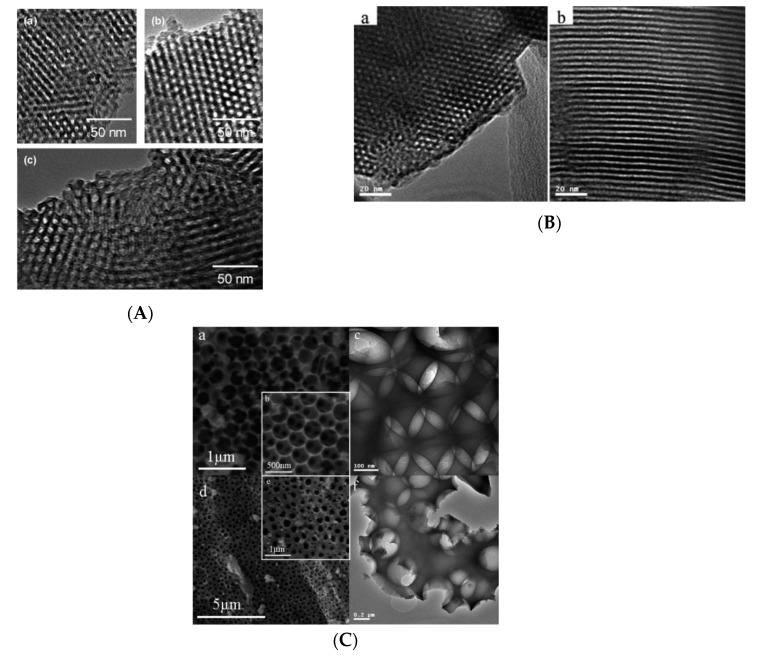
(**A**) TEM images of calcined aluminum methylene diphosphonates prepared in the presence of: (a) EO_80_PO_30_EO_80_ (Pluronic F68), (b) EO_106_PO_70_EO_106_ (Pluronic F127), and (c) EO_20_PO_70_EO_20_ (Pluronic P123) tri-block co-polymers, respectively. Reprinted with permission from [75]. Copyright (2005), American Chemical Society. (**B**) (a,b) TEM images of periodic mesoporous titanium phosphonate-1 hybrid material. Reprinted with permission from [85]. Copyright (2010), The Royal Society of Chemistry and (**C**) (a,b) SEM and (c) TEM images of titanium phosphonate hybrid; (d,e) SEM and (f) TEM images of titanium phosphonate hybrid with β-cyclodextrin. Reprinted with permission from [82]. Copyright (2008), American Chemical Society.

Templated mesoporous aluminum phosphonate can be synthesized via the reaction of aluminum isopropoxide and methylene diphosphonic acid with octadecyltrimethylammonium chloride (ODTMACl) as the SDA in the presence of tetramethylammonium hydroxide. The hybrid structure had an amorphous network with surface area of 738 m^2^/g and a pore size of 1.8 nm. The pores were periodically arranged in a 2D hexagonal mesophase as analyzed by low-angle XRD and TEM. However, these materials were soluble in acidic ethanol solutions and had to be calcined in order to remove the surfactant and enhance condensation, which caused the loss of periodicity of the porous network in the final material [72]. Another method to produce mesoporous aluminum diphosphonates was by the “atrane route” where aluminum butoxide is heated with nitrilotriethanol to obtain aluminum atrane complexes and subsequently reacted with ethylene diphosphonic acid in the presence of CTAB as the SDA. This hybrid mesoporous material had a surface area of 674 m^2^/g with a pore size of 3.3 nm. These materials were formed on the basis of S^+^ I^−^ ionic self-assembly, leading to the creation of a mesoporous aluminum diphosphonate [73,74]. Some examples of other aluminum diphosphonates based on the atrane route protocol in the presence of different templates such as F127, P123, Brij-56 and F68 are shown in Figure 6A [75,76].

Interestingly, mesoporous metal phosphonates have also been synthesized with the use of monophosphonates. The synthesis of tin phenylphosphonate in the presence of sodium dodecyl sulfate (SDS) as the template led to the formation of a material that displayed microporosity and mesoporosity, exhibiting a combined type-I and type-IV isotherm. This material was built-up from a layered structure consisting of tin (IV) phosphonate nanoparticles with microporosity. During calcination, thermal shrinkage resulted in the reduction in the inter-particle voids that were between 5 and 8 nm [77]. When the choice of metal was altered to zirconium and reacted with phenyl phosphonic acid in the absence of any supramolecular templates, a mesoporous material was obtained. The origin of the mesoporosity was attributed to the formation of a hydrophobic core by the phenyl groups, which led to the formation of pores with sizes of 5–10 nm [78]. Unique phosphonic linkers such as 1-phosphonomethylproline (H_3_PMP) have been utilized with the purpose of synthesizing mesoporous aluminum phosphonates whose pores were lined by chiral amino acids. The atrane route was utilized to prepare this hybrid material, which possessed a surface area of 422 m^2^/g and a pore size of 3.7 nm [79]. Another methodology that was followed to obtain mesoporous metal phosphonates without the use of templates was based on the use of a complex tetraphosphonic linker that prevented the formation of layered motifs and made it possible to achieve open structured hybrid metal phosphonates. Tetrakis-1,3,5,7-(4-phosphonatophenyl) adamantane (TPPhA) was one such complex phosphonic linker, which upon non-hydrolytic condensation with titanium and vanadium precursors, resulted in the formation of a hybrid mesoporous material with a surface area of 550 and 118 m^2^/g, respectively [80,81].

Mesoporous metal phosphonates have also been synthesized by using polyphosphonic acids that feature a hetero-atom bridging the two or more phosphonate units. The hybrid materials synthesized with this strategy allowed to prevent the formation of a lamellar morphology and obtain interesting porous architectures. The 1-hydroxy ethylidene-1,1-diphosphonic acid (HEDP), a diphosphonate containing a hydroxy group in the bridging unit of the diphosphonic acid, was reacted with titanium butoxide in the presence/absence of β-cyclodextrin and polystyrene (PS) spheres by a hydrothermal method. This material showed no porosity apart from being macroporous due to PS spheres as shown in Figure 6C. This resulted in a low surface area around 23–25 m^2^/g [82]. A higher surface area was achieved by reacting the same inorganic and phosphonic acid reagents under milder synthesis conditions (room temperature) and in the absence of the PS spheres and β-cyclodextrin. The result was a hybrid porous titanium phosphonate that showed a combination of a type-II and type-IV isotherm, with a surface area of 158 m^2^/g. The texture of the material showed inter-particle voids with a layered structure as observed by electron microscopy [83]. However, the incorporation of soft templates like pluronic templates (F127/P123) in the condensation reaction of HEDP and titanium butoxide at room temperature, led to a material with a larger surface area of ~377–511 m^2^/g, respectively. The results from the nitrogen sorption isotherm and SEM/TEM implied that the texture of the material was arising from inter-particle voids in the mesopore and macropore region [84]. To enhance the surface area further, claw type polyphosphonic acids containing hetero-atoms with titanium precursors can be used, resulting in materials that form hybrid networks with pores containing unreacted P–OH groups, as shown by Figure 6B [85]. When reacting titanium tetrachloride and ethylenediamine tetramethylene phosphonic acid (EDTMP) in the presence of Brij-56 as the template, a hybrid titanium phosphonate having hexagonally ordered mesopores (2–3 nm in size) with type-IV isotherm and a surface area ~1000 m^2^/g was obtained. By changing the SDA from Brij-56 to CTAB, a tunable mesopore assembly ranging from a hexagonal phase to a cubic phase was achieved [86]. Moreover, template-free methods have been applied in which EDTMP was reacted with titanium butoxide, resulting in the formation of a porous material with hierarchical inter-particle porosity [87,88].

New concerted efforts in constructing hybrid porous metal phosphonates have been directed towards achieving control over the hetero-condensation of metal and phosphonic precursors. Recently, porous aluminum phosphonate synthesized with partially hydrolyzed acidic intermediates, formed from pre-hydrolyzed bisphosphonate ester precursors were reported. The partial hydrolysis allows to tune the reactivity towards aluminum chloride [89]. This synthesis methodology allowed the incorporation of a variety of organic linkers such as amides, thiophene, and ether moieties into porous metal phosphonate networks [90]. Another novel approach has been utilizing the non-hydrolytic sol–gel methodology to synthesize hybrid titanium phosphonate by the use of biphenyl bisphosphonate ester and titanium isopropoxide in acetic anhydride. These materials showed titanium oxide nanoparticles that were crosslinked with the bisphosphonate esters creating a high surface area material of 830 m^2^/g [91]. 

## 3. Applications 

Porous hybrid metal phosphonates show great potential in applications covering a wide diversity of domains. In this section, however, we will discuss those key application areas that we believe benefit most from the synergic combination of the organophosphonate functionality and the metal units. We will focus on those materials with high specific surface areas induced by a porous network, enabling the possibility of creating a tunable interface between the substrate (hybrid material) and the reactive/interactive species. Tailorable materials with surface-specific functionalities are vital in applications such as: (i) separation/extraction processes, (ii) heterogeneous catalysis and (iii) proton conduction. These areas are the target domains wherein porous metal phosphonates would provide a good choice and hence we focus on these fields here.

### 3.1. Separation and Extraction

A summary of the application of porous metal phosphonates in separation and extraction are displayed in Table 1. An example is the mesoporous hybrid system based on aluminum with BHMT (N-based pentaphosphonic) and ATMP (N-based triphosphonic) phosphonate moieties in the presence of F127 as a surfactant, providing a hierarchical meso/macroporous texture (type-II isotherm) with inter-particle mesoporosity and surface areas of 128 and 154 m^2^/g, respectively. They were evaluated for the extraction of copper ions from an aqueous solution (10–50 mg/L). The adsorption of Cu^2+^ ions (10 mg/L) resulted in a K_d_ (distribution coefficient) of 277,485 and 17,281 mL/g for BHMT and ATMP aluminum phosphonate hybrids, respectively, at a pH ~6–8. However, with higher Cu^2+^ concentrations (50 mg/L) the K_d_ value decreased significantly to 6370 mL/g and 2744 mL/g for BHMT and ATMP aluminum frameworks, respectively. The adsorption was attributed to the copper chelation to nitrogen and RPO_3_^2−^ groups present in the hybrid framework and their K_d_ (at lower Cu^2+^ concentration) was comparable to mesoporous silica [92,93]. Next to metal adsorption, the same materials were applied for the adsorption of large size molecules such as proteins, in this example lyzozyme. The hybrid frameworks followed Langmuir type adsorption with a monolayer capacity of 11.8 µmol/g (pH = 11), 10.42 µmol/g (pH = 12), 9.23 µmol/g (pH = 9.6), and 8.24 µmol/g (pH = 6.5). The maximum adsorption capacity was observed at the isoelectric point of the protein (pI = 11). Therefore, the hydrophobic interactions between the protein molecules and the alkyl chains lining the pores of the hybrid BHMT–aluminum phosphonate are promising for development of adsorbents for large molecule-like proteins [94]. However, these hybrid materials display lower uptake in comparison to mesoporous silica (>100 µmol/g) such as SBA-15 and PMOs [95,96,97,98]. 

A hybrid framework based on zirconium phosphonate created from zirconium(IV) precursors and ATMP has been evaluated as cation-exchange adsorbent. This hybrid material was chosen for the separation of three groups of metals; transition metals (Cu^2+^, Zn^2+^, Ni^2+^ and Co^2+^), heavy metals (Pb^2+^, Hg^2+^, and Cd^2+^) and f-block metals (Ce^3+^ and Th^4+^). The preferential order of adsorption amongst the transition metals were Cu^2+^ > Ni^2+^ > Co^2+^ > Zn^2+^ (K_d,Cu_ = 9879.7, K_d,Ni_ = 1550.0, K_d,Co_ = 600.0, and K_d,Zn_ = 584.6 mL/g), for heavy metals Pb^2+^ > Cd^2+^ > Hg^2+^ (K_d,Pb_ = 1261.5, K_d,Cd_ = 1037.5, and K_d,Hg_ = 56.4 mL/g), and for f-block metals Ce^3+^ > Th^4+^ (K_d,Ce_ = 2000.0, and K_d,Th_ = 114.7 mL/g) in aqueous solution. However, in acetic acid, HNO_3_, and ammonium nitrate solutions, the distribution coefficients were reduced due to ion competitions. Amongst the metal ions studied, only Cu^2+^ (in aqueous, 0.02 and 0.2 M acetic acid) and Ce^3+^ (in 0.02 M acetic acid) showed complete sorption with a high separation factor (SF) between Cu^2+^/Zn^2+^ (16.9) and Ce^3+^/Th^4+^ (17.54) [99]. Similarly, hybrid manganese phosphonate synthesized from MnCl_2_ and EDTMP was evaluated for metal ion uptake and protein adsorption. The optimal pH for the Cu^2+^ uptake was ~6 and the adsorption followed Langmuir behavior with a maximum monolayer capacity of 79.5 mg/g at 20 °C. This value is in the same order of magnitude as that of mesoporous silica adsorbents (~24.8 mg/g) and hybrid titanium phosphonates (also made with EDTMP showed ~28.8 mg/g uptake) [100].

**Figure 7:**
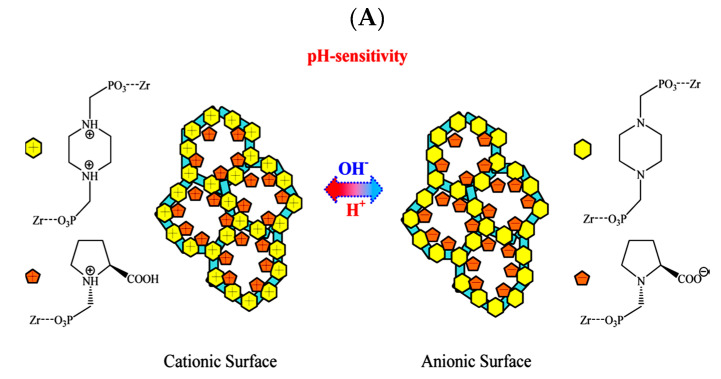

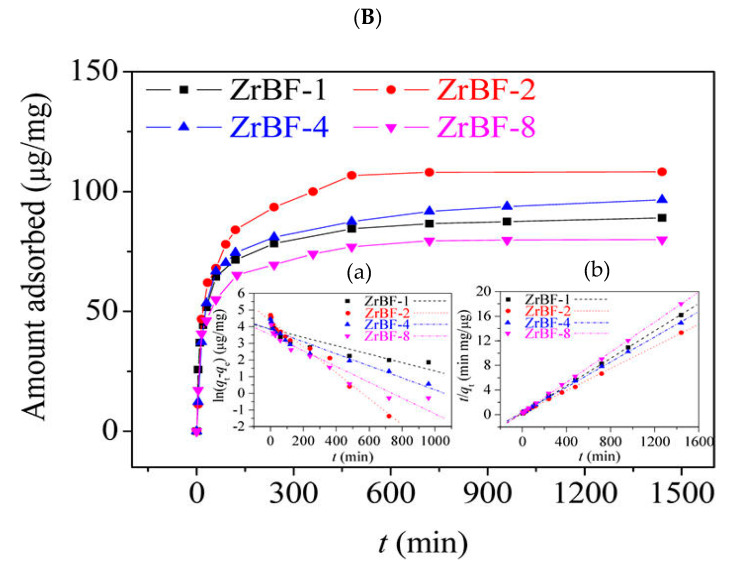
(**A**)—illustrative scheme for ZrBF’s biofunctionality, pH sensitivity, and functionalizability; (**B**)—adsorption kinetics of ZrBF-n (n = 1, 2, 4 and 8) for DNA. Insets—linear fit according to the pseudo-first order rate equation (a) and pseudo-second order rate equation (b), respectively. Reprinted with permission from [101]. Copyright (2013), American Chemical Society.

Zirconium hybrid phosphonate frameworks made from 1-phosphomethylproline (H_3_PMP) and 1,4-bis(phosphomethyl) piperazine (BPMP) phosphonic ligands resulted in a pH-sensitive porous framework. This framework was employed for the adsorption and delivery of nucleic acids without further modification/post-treatment. The adsorption of salmon sperm DNA onto the framework followed Langmuir type adsorption. Among the various hybrids created by this method, the ZrBF-2 showed the largest equilibrium monolayer capacity of 238.6 mg/g. The increased uptake was attributed to the presence of uncondensed P–OH groups and pyridinic N-atoms present along the porous framework of the hybrid material [101]. This uptake by the hybrid framework is comparable to salmon sperm DNA uptake of 375 and 110.7 mg/g by magnetic mesoporous silica adsorbents [102,103]. The mechanism of adsorption followed pseudo-second order kinetics. Moreover, the framework was pH responsive, allowing adsorption–desorption cycles by changes in the pH. The structure exhibited a cationic surface under acidic and neutral pH where the surface could adsorb negatively charged DNA molecules by electrostatic interactions. Subsequently, the release of the nucleic acid was triggered by increasing the pH, changing the surface charge and resulting in desorption. This allowed for the pH controlled loading and unloading of nucleic acids as illustrated by Figure 7 [101].

In another application, zirconium phosphonate frameworks synthesized by zirconium(IV) and phenyl phosphonic acid in the presence of an SDS template were employed as immobilized metal affinity chromatography (IMAC) for the selective enrichment of phosphopeptides. The specificity for phosphopeptides was evaluated with use of a tryptic digest consisting of a mixture of bovine serum albumin (BSA) and bovine-β-casein (B-β-C) as the sample peptides (mass ratio B-β-C:BSA = 1:5). The specificity of the bovine-β-casein adsorption was attributed to the chelation of phosphopeptide to the Zr(IV) metal centers present in the framework [104]. 

Finally, we shall go through the use of porous metal phosphonates for the solid-phase extraction of lanthanides and actinides. Tin phosphonate synthesized from various polyphosphonic acids (HEDP, ATMP, EDTMP and DTPMP) was employed for radionuclide solid-phase separation. The study consisted of: a batch type sorption of metal ions Cs^+^, Sr^2+^, Al^3+^, Fe^3+^, Th^4+^, UO_2_^2+^, and rare earth elements (REE) (all lanthanides) in 0.1, 1 and 3 M HNO_3_ solutions and a competitive sorption kinetics study for the metal ions Eu^3+^, Fe^3+^, La^3+^, Lu^3+^, Sr^2+^, Th^4+^, and UO_2_^2+^ in 1M HNO_3_ solution. In the 0.1 M HNO_3_ solution, tin phosphonate hybrids containing HEDP displayed a high uptake of middle lanthanides (K_d_ > 6000 mL/g), whereas the EDTMP displayed a high uptake of late lanthanides (K_d_ > 4000 mL/g), showing the importance of the type of phosphonic linker. However, a high uptake of Fe^3+^ (K_d_ ~1000–10,000 mL/g) was observed among all the hybrids in 0.1 M HNO_3_ solution. An interesting observation was that all the hybrid materials showed extreme affinity for the Th^4+^ radionuclide in all nitric acid solutions (K_d_ ~127,171 mL/g and 164,399 mL/g for ATMP and EDTMP hybrids). Nevertheless, although these hybrids showed promising capabilities in use as a solid-phase adsorbent, they displayed slow kinetics for the uptake of radionuclides [105]. 

Mixed hybrids of zirconium phosphonate–phosphate (phosphonate/phosphate ratio from 1.5:1 to 1:8) were evaluated for solid-phase lanthanide extraction from an aqueous mixture of metal ions. In this study, the choice of metal ions were Nd^3+^, Sr^2+^, and Cs^+^. The Nd^3+^ ions were always adsorbed onto the hybrid materials >98% at all phosphate ratios in the presence of Sr^2+^ and Cs^+^ ions. The uptake capacity increased with higher phosphate amounts for Nd^3+^ but at very high phosphate amounts, the uptake of Sr^2+^ and Cs^+^ ions increased, and led to a lower separation factor (SF) with respect to Nd^3+^ ions [106]. In the same study, hybrid tin phosphonate–phosphate were compared with a Zr(IV) phosphonate–phosphate system. The Zr-hybrid showed clear affinity only towards M^3+^ ions, whereas the Sn-hybrid showed affinity to M^3+^ and slightly for M^2+^ ions. Thus, showing the importance of the choice of metal and phosphonate/phosphate ratio while creating hybrid material used for the solid-phase extraction of lanthanides [106]. 

In another study, Zr/Sn–phosphonates containing no inorganic phosphate were also evaluated for selective separation. These were subjected to the lanthanide extraction of Nd^3+^, Eu^3+^, and Ce^3+^ in the presence of Cs^+^ and Sr^2+^ at pH = 3. Additionally, here, the Zr-based hybrid phosphonates showed the preferential uptake of only +3 metal ions, whereas the Sn-based hybrids had a slight affinity towards Sr^2+^ along with +3 metal ions. These hybrids were also evaluated for the removal of Am^3+^ from spent nuclear fuel. Both Zr and Sn hybrids showed >99% removal of Am^3+^ from the solution (pH = 3) with K_d_ of 78,000 and 30,000 mL/g, respectively. To evaluate the radiolytic stability of these hybrid materials, the sorbents were subjected to an irradiation with a Co-60 gamma source 3.18 × 10^6^ Gray at a dose of 1.617 × 10^5^ rad/hr. The hybrid materials retained their structure as evidenced by XRD and showed a similar uptake of Am^3+^ ions after being irradiated [107].

**Figure 8:**
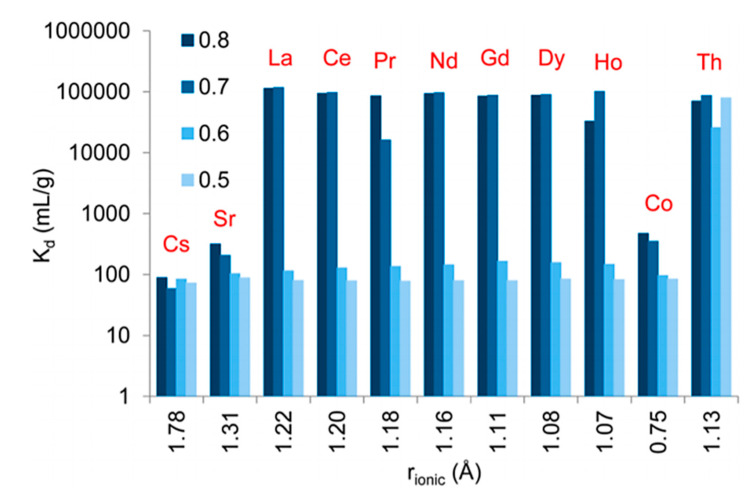
Extraction of cations of different ionic radius by poorly crystalline ZrBTP-0.8, ZrBTP-0.7, ZrBTP-0.6, ZrBTP-0.5, prepared in the absence of HF from mixed cation solutions with each cation at nominal 20 mg/L in 0.10 M HNO_3_. Reprinted with permission from [108]. Copyright (2016), American Chemical Society.

Zirconium phosphonate were synthesized hydrothermally, using the benzene-1,3,5-triphosphonic acid (BTP) ligand to obtain both amorphous and crystalline (in the presence of HF) frameworks. These materials were evaluated for metal separation from a mixed solution containing the following ions: Cs^+^, Sr^2+^, La^3+^, Ce^3+^, Pr^3+^, Nd^3+^, Gd^3+^, Dy^3+^, Ho^3+^, Co^2+^ and Th^4+^ with an ionic strength of 1 mmol/L and the nominal concentration of individual ions ~20 mg/L in 0.1 M HNO_3_ solution. Figure 8, shows the extraction of the different ions as a function of ionic radii of the metal ions by the hybrid Zr-phosphonate in function of the phosphate/zirconium ratio in the material varying from 0.5 (low) to 0.8 (high). The order of the selectivity of ions followed: Th^4+^ > Ln^3+^ > Co^2+^, Sr^2+^ > Cs^+^, and the selectivity was dependent on the cation valence and polarizability. With higher molar amounts of phosphonic ligands, the K_d_ increased, suggesting that the binding of the cations is achieved via the phosphonic acid groups distributed in the framework. A competitive kinetics study showed that at equilibrium (concentration of metal ions > 1 mg/L), Dy^3+^ and Ho^3+^ were strongly extracted in mixed cation solution containing the rest of the (REE) ions along with Cs^+^, Sr^2+^ and Co^2+^. The authors highlighted in the same work that the materials that were amorphous and free of fluoride performed with enhanced selectivity and better kinetics than the crystalline materials in highly acidic solutions. This study showed that the distribution of unreacted phosphonic acid moieties was one of the important criteria for improved cation binding to the hybrid framework [108].

In another study, porous adsorbents based on zirconium phosphonate have been prepared hydrothermally with ZrCl_4_/ZrOR_4_ and ATMP. These hybrid sorbents were evaluated for competitive lanthanide separation from a mixture of cations. All zirconium phosphonate hybrids showed a selective uptake of lanthanides over Cs^+^, Sr^2+^ and Co^2+^. Among the lanthanides, Eu^3+^ was the most strongly sorbed lanthanide across all the hybrids. Interestingly, the amorphous hybrids in this study also displayed a higher uptake compared to the crystalline hybrid materials. The presence of fluoride ions had a detrimental effect on the uptake of lanthanide ions resulting in the lower capacity presented by the crystalline hybrids. Finally, the choice of metal precursor was evaluated, the zirconium chloride-based hybrids led to materials that displayed better lanthanides (>99% extraction of La^3+^, Nd^3+^, Eu^3+^, Ho^3+^ and Yb^3+^) uptake than the materials prepared from zirconium isopropoxide (~40–70% extraction of La^3+^, Nd^3+^, Eu^3+^, Ho^3+^ and Yb^3+^). However, the zirconium isopropoxide-based hybrids showed more selectivity among the lanthanides over Cs^+^ and Sr^2+^. The changes in sorption properties were attributed to the differences in the incorporation of the phosphonate moiety (ATMP) into the hybrid network [109].

**Table 1:** Separation and extraction application list for porous metal phosphonates.

Metal Precursor	Phosphonic Linker	Synthesis	Application—Separation/Extraction	Reference
Al(O^s^Bu)_3_	ATMP BHMT	Hydrothermal	Cu^2+^ adsorption and lyzozyme adsorption	[94]
ZrOCl_2_	ATMP	Precipitation	Cation exchange—divalent metal ions	[99]
ZrCl_4_	H_3_PMP BPMP	Hydrothermal	Adsorption and delivery of DNA	[101]
ZrCl_4_	PPA	Hydrothermal	Immobilized metal affinity chromatographic adsorbent for phosphopeptide enrichment	[104]
MnCl_2_	EDTMP	Hydrothermal	Cu^2+^ sorption and selective protein adsorption of Cyt–C over BSA	[100]
SnCl_4_	HEDP ATMP EDTMP	Hydrothermal	Radionuclide separation of Th^4+^	[105]
ZrOCl_2_	BTP BDP BMP	Hydrothermal	Lanthanide and actinide separation	[108]
ZrOCl_2_	BDPA H_3_PO_4_	Hydrothermal	Ion-exchange metal ions	[106]
ZrOCl_2_ SnCl_4_	BDPA H_3_PO_4_	Hydrothermal	Actinides separation	[107]
ZrCl_4_/Zr(O^i^Pr)_4_	ATMP	Hydrothermal	Lanthanides extraction	[109]

Abbreviation: ATMP = aminotris(methylene phosphonic acid); BHMT = bis(hexamethylene-triamine)-penta(methylene phosphonic acid); H_3_PMP = 1-phosphomethylproline; BPMP = 1,4-bis(phosphomethyl) piperazine; PPA = phenyl phosphonic acid; EDTMP = ethylenediamine tetra(methylene phosphonic acid); HEDP = 1-hydroxyethane 1,1-diphosphonic acid; BTP = benzene 1,3,5-triphosphonic acid; BDP = 3,5-diphosphonobenzoic acid; BMP = 5-phosphonoisophthalic acid; BDPA = 1,4-phenylenediphosphonic acid.

### 3.2. Catalysis

A list of the catalytic applications for which metal phosphonates have been used for is summarized in Table 2. As an example, mesoporous vanadium phosphonate has been employed as catalyst for the oxidation of benzyl alcohols. The oxidation reaction resulted in high conversion (>99%) and selectivity (96%) in the formation of benzaldehyde. There was no formation of side reaction products such as benzoic acid or ethers due to the over-oxidation or acid condensation, respectively. Benzyl alcohols containing electron-donating groups (4-MeOPhCH_2_OH and 4-MePhCH_2_OH) and electron withdrawing groups (4-NO_2_PhCH_2_OH and 4-CF_3_PhCH_2_OH) were also successfully converted to the respective aldehydes with similar conversion and selectivity as in the case of benzaldehyde. Evidence of some shape selectivity was observed, as 2,4,6-trimethyl benzyl alcohol oxidation resulted in poor conversion (56%) and low selectivity (79%) compared to other benzyl alcohol substrates [81].

One of the benefits of hybrid inorganic–organic materials is that they can introduce chirality in the material’s porous network through the organic moiety on the phosphonate linker. The hydrolysis of styrene oxide under confined homochiral environment available in the hydrophobic pores of hybrid titanium phosphonate containing leucine moieties was evaluated. The yield of the reaction in the presence of the hybrid catalyst was 30% S-styrene oxide with an enantiomer efficiency (EE) of >99%. Moreover, other epoxides such as 4-methyl styrene oxide and α-4-methyl styrene oxide reacted in an identical manner, with a yield of ~20% having ~98% and ~95% EE, respectively. The authors postulated that the epoxide was activated by moieties having a partial positive charge in the titanium phosphonate framework such as the phosphonate, or Ti(IV) centers, followed by the attack of water directed by the homochiral leucine peptide to yield S-styrene glycol [110]. Similarly, the enantioselective addition reaction to obtain optically active phenylpropanol has also been catalyzed by hybrid titanium phosphonates [111]. 

Zirconium phosphonates were employed as a solid-acid catalyst in the synthesis of methyl-2,3-o-isopropylidene-β-d-ribofuranoside. The hybrid material was compared alongside commercial acidic resin, NKC-9 and concentrated hydrogen chloride. The reaction was carried out at 70°C and yielded 28% after 2 h when using the hybrid zirconium phosphonate, which levelled off at 35.6% after 3 h. With the commercial resin NKC-9, the yield of 21.1% and 33% was reached after 2 h and 3 h of reaction, respectively. Thus, the hybrid zirconium phosphonate performed similar to the commercial resin even though the resin had substantially higher proton concentrations [112]. In another example, porous zirconium phosphonates were evaluated as a solid-acid catalyst for the esterification of acetic acid with cyclohexanol as well as for the hydrolysis of ethyl acetate in aqueous media. The results were compared with commercial resin NKC-9 and ZrPO_4_. After 10 h of reaction between acetic acid and cyclohexanol at 100 °C, the NKC-9 showed the highest yield of 87.9% followed by the zirconium hybrid phosphonates synthesized with HEDP, ATMP, and EDTMP phosphonic acids providing yields of 76.8%, 65.4% and 75.3%, respectively. The inorganic ZrPO_4_ gave a yield of 72.9% under similar reaction conditions. However, the TOF (measured at 100 °C) for the hybrid zirconium phosphonates based on EDTMP, HEDP and ATMP were 310.2, 234.0 and 306.1, respectively. These TOF were higher than those of the commercial resin, NKC-9 (118.7) and the inorganic ZrPO_4_ (210.1). As a side note: these reported TOF values were calculated based on the products formed, hence their activity calculation also includes the occurring diffusion/adsorption effects that might be rate determining. Moreover, the authors seem to have calculated turnover number (TON) according to the definition of Kuzoch et al., 2012 rather than TOF values [113]. The acid content of the hybrid zirconium phosphonates was 1.92, 1.25, and 1.42 mmol/g for the HEDP, ATMP and EDTMP-based hybrids (NKC-9 = 4.33 and ZrPO_4_ = 2.03 mmol/g), respectively. The higher TOF values for the hydrolysis reaction in the presence of hybrid zirconium phosphonates was attributed to higher specific surface areas and the surface hydrophobicity of the catalyst. When the same hydrolysis reaction of ethyl acetate was performed in aqueous media, the catalytic activity per acidic proton was 51.1, 21.5, 30.6, 35.2, and 15.1 mmol/mol_acid_/min for Zr-EDTMP, Zr-HEDP, Zr-ATMP, NKC-9 and inorganic ZrPO_4,_ respectively [114]. 

In another example, hybrid iron (III) phosphonate, created by the reaction of iron (III) chloride with BTP precursors, was employed as a solid-acid catalyst for transesterification reactions. Amongst many reactants, ethyl cyanoacetate showed the highest conversion of 88.3% with a selectivity of 100% (at 60 °C, 6 h). It was observed that as the electron withdrawing effect (CN > C(=O)CH_3_ > Cl) of the ester reactant decreased, the conversion to the product decreased. The electrophilicity of the carbonyl carbon of the ester reactant was attributed as the main driving force for the reaction. Negatively charged free phosphonic acids in the framework of the hybrid material could prevent molecules enriched with p-electron clouds entering the porous channels. Thus, providing the catalyst with selectivity for the transesterification of esters that contain fewer mobile electrons [115].

**Figure 9:**
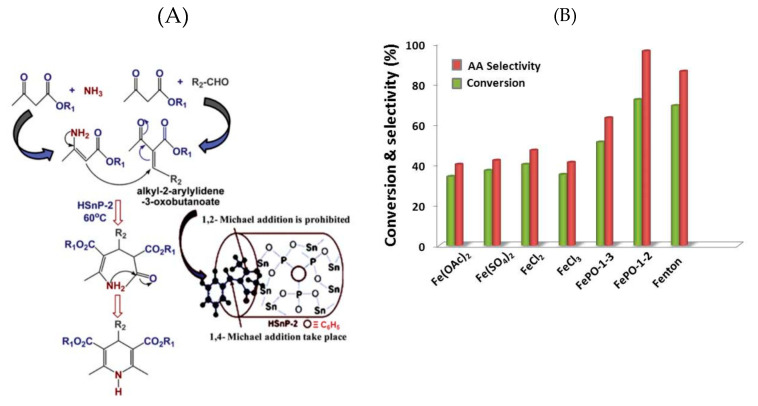
(**A**)—probable reaction mechanism and the role of the HSnP-2 catalyst in selective synthesis of 1,4-dihydropyridines. Reprinted with permission from [117]. Copyright (2013), The Royal Society of Chemistry and (**B**)—dependence of the product yield on various catalysts for the aerobic oxidation of cyclohexanone at 75 °C. Reprinted with permission from [120]. Copyright (2016), American Chemical Society.

Next to the phosphonate moiety discussed in the previous paragraph, the organic group and metal center can also play an important functional role in catalysis. Porous tin phosphonate made from tin(IV) chloride and pentaethylenehexamine–octakis-(methylphosphonic acid) hexadecenoic sodium was employed as Baeyer–Villiger catalyst for the oxidation of cyclohexanone in the absence of peroxides as an oxidant. The product of this reaction was the formation of adipic acid with a yield of ~74%. When the hybrid material was replaced with an inorganic tin(IV) phosphate, the yield was only of the order ~22%, suggesting the key role played by the organic group providing free –NH_2_ groups in the porous framework, aiding in stabilizing the keto-enol tautomerization. In addition, the enol form is also stabilized by the Sn-centers. Furthermore, the Sn-center was found to activate the molecular oxygen and helped to form the cyclic six-membered transition state compound that later rearranged to form a cyclic ester as a second intermediate [116]. In another example, hybrid tin(IV) phosphonates synthesized from tin (IV) chloride and BTP were used as catalysts for the synthesis of 1,4-dihydropyridines from 1,3-dicarbonyls and ammonium acetate under solvent-free conditions (Hantzsch ester reaction). It was observed that the 1,4-addition was selectively facilitated over the 1,2-addition to the unsaturated C=O compound in the presence of the hybrid tin(IV) phosphonate catalyst. The authors attributed this to the adsorption of unsaturated C=O to the hydrophilic surface of the porous framework containing Sn-centers. Since there were no free C=O groups, the enamine exclusively attacked the C=C bond resulting in the formation of only 1,4-dihydropyridine as illustrated in Figure 9A [117]. Alternatively, porous molybdenum(V) phosphonate made with MoCl_5_ and BTP was employed as a catalyst for the one-pot synthesis of 2-aryl-benzimidazoles at room temperature by the condensation of o-phenylenediamine and substituted aromatic aldehydes (yield for 2-phenyl benzimidazole = 93%). When the same reaction was catalyzed by pure H_3_PO_4_ and MoCl_5,_ lower yields of 53% and 40%, respectively, were obtained. Therefore, the presence of vacant d-orbitals of the Mo metal centers along with the free P–OH groups were correlated to the activation of the C=O group of the aldehydes [118].

Fenton-like heterogeneous catalysts from hybrid cobalt phosphonates, made from CoCl_2_ and DTPMP, were employed for the catalytic oxidation of methylene blue (MB). The optimal pH for the degradation reaction was around ~7, and the complete degradation of MB occurred within 25 min, with a degradation rate of 0.136 min^−1^. Decreasing the pH to 5 and 2 resulted in a notable deterioration of the MB degradation rate of 0.0331 and 0.0203 min^−1,^ respectively. Similarly, at higher pH ~9, the degradation reduced to a rate of 0.0931 min^−1^. Under acidic conditions, electrostatic repulsion between the positively charged hybrid framework and MB molecules prohibited effective adsorption. Under alkaline conditions, the lower degradation was attributed to the fact the sulfate radicals generated in the reaction system were quenched by the OH^−^ ions and possibly also by the formation of Co(OH)_2_ that might form on the surface of the hybrid cobalt phosphonates [119]. In another example, hybrid iron phosphonate was employed as a Fenton-like catalyst for the catalytic oxidation of cyclohexanone to adipic acid in an aqueous medium at 75 °C for 10 h under oxygen atmosphere and in the absence of peroxide. The conversion to adipic acid was determined to be 72% with a selectivity of 96%. The hybrid iron phosphonate catalyst had comparable results to other Fe-based catalysts with the important difference that other classical heterogeneous Fenton catalysts required hydrogen peroxide as the oxidant (Figure 9B). The authors explained that the Fe(II) sites are responsible for the activation of molecular oxygen, in contrast to the strong oxidizing capability of Fe(III) [120].

In a later study, the above hybrid materials were evaluated as catalysts for the cycloaddition of CO_2_ with aziridines to obtain oxazolidinones at 120 °C under 3 MPa pressure of CO_2_ for 6 h under solvent-free conditions. The three zirconium phosphonate hybrids showed a high conversion of >98% and regioselectivity with yields of 85%, 79% and 90% for Zr-HEDP, Zr-ATMP, and Zr-EDTMP, respectively. Among these three hybrid catalyst systems, the Zr-HEDP displayed the highest regioselectivity of 96:4 in the formation of 3-ethyl-5-phenyloxazolidin-2-one with respect to 3-ethyl-4-phenyloxazolidin-2-one, in comparison to the Zr-ATMP (95:5) and Zr-EDTMP (90:10) hybrids. The authors proposed a mechanism wherein the proton from the -P-OH group coordinated with the N-containing aziridine and the adsorbed CO_2_ interacted with the -OH group of the HEDP moiety. This way, the aziridine was attacked by the activated CO_2_ (nucleophilic), leading to the formation of carbamate, followed by intramolecular ring closure resulting in oxazolidinone formation [121].

**Table 2:** Summary of the porous metal phosphonates for catalytic application.

Metal Precursor	Phosphonic Linker	Synthesis	Application—Catalysis	Reference
V(O)(*i*-PrO)_3_	TPPhA	Non-hydrolytic condensation	Oxidation of benzylic alcohols	[81]
Ti(SO_4_)_2_	(1R, 2S)-(-)-2-P (1R, 2S)-(+)-2-P	Precipitation (Hydrothermal)	Enantioselective addition of benzaldehyde	[110]
Ti(O^i^Pr)_4_	Polypeptide capped with phosphonic acid moiety	Non-hydrolytic condensation	Enantioselective hydration of styrene oxide	[111]
ZrCl_4_	HEDP	Precipitation/Hydrothermal	Solid-acid catalyst for synthesis of methyl-2,3-*O*-isopropylidene-β-D-ribofuranoside	[112]
SnCl_4_	PEHMP	Precipitation/Hydrothermal	Oxidation of cyclohexanone to adipic acid in absence of peroxides	[116]
FeCl_3_	BTP	Precipitation/Hydrothermal	Transesterification for synthesis of biofuels	[115]
SnCl_4_	BTP	Precipitation/Hydrothermal	Synthesis of 1,4-dihydropyridines	[117]
MoCl_5_	BTP	Precipitation/Hydrothermal	Synthesis of benzimidazoles	[118]
CoCl_2_	DPTMP	Hydrothermal	Catalytic oxidation of methylene blue	[119]
ZrOCl_2_	HEDP ATMP EDTMP	Precipitation/Hydrothermal	Hydrolysis of ethyl acetate; esterification of acetic acid with ethanol and cyclohexanol	[114]
ZrOCl_2_	HEDP ATMP EDTMP	Precipitation/Hydrothermal	Cycloaddition of aziridnes and CO_2_	[121]
FeCl_3_	HEDP	Precipitation/Hydrothermal	Oxidation of cyclohexanone to adipic acid	[120]

Abbreviation: TPPhA = tetrakis-1,3,5,7-(4-diethylphosphonatophenyl)adamantane; (1R,2S)-(-)-P = (1R,2S)-(-)-2-[N-(diethylethylphosphosphonate)]imino-1,2-diphenylethanol; (1R, 2S)-(+)-P = (1R,2S)-(+)-2-[N-(diethylethylphosphosphonate)]imino-1,2-diphenylethanol; HEDP = 1-hydroxyethane 1,1-diphosphonic acid; PEHMP = pentaethylenehexamine-octakis-(methylphosphonic acid); BTP = benzene 1,3,5-triphosphonic acid; DTPMP = diethylenetriamine penta(methylenephosphonic acid); ATMP = aminotris(methylene phosphonic acid); EDTMP = ethylenediamine tetra(methylene phosphonic acid).

### 3.3. Proton Conduction

As a final application in which porous metal phosphonates can have a particular benefit, proton conduction is discussed. A summary of the proton conducting frameworks has been listed in Table 3. The framework built-up by zinc precursor and BTP as the phosphonate ligand (denoted as PCMOF-3) was evaluated for proton conduction. The aquo ligands on zinc were ascribed as the main source of protons to be transported. The proton conductivity of the framework was strongly dependent on the humidity, with 4.5 × 10^−8^ S/cm at 44% RH (25 °C) and 1 × 10^−5^ S/cm at 98% RH (25 °C). The activation energy (E_a_) for the proton transfer ~0.17 eV was derived from the bulk conductivity of the PCMOF-3 framework. In comparison, the activation energy for 1 mol/L HCl has been reported as 0.11 eV and that of Nafion as 0.22 eV. Thus, PCMOF-3 represents a proton-conducting metal phosphonate framework in which the conductivity does not involve secondary SO_3_H sites. The polar interlayer of the framework serves as a scaffold for highly ordered water molecules that form a Grotthuss proton transfer pathway, as evidenced by the low E_a_. The study concluded with the evidence that both the phosphonate ‘O’ atoms and Zn aquo ligands contributed to the proton transfer process [122].

In another study, the β-PCMOF-2 framework, prepared by sodium and phloroglucinol in the presence of chlorosulfonic acid was applied. This sulfonate framework displayed a proton conductivity of 1.3 × 10^−3^ S/cm at 85 °C and 90% RH. In order to improve the conductivity, an isomorphic ligand exchange was performed with a BTP ligand to replace part of the aryl sulfonic moieties with the BTP ligands, forming a new mixed PCMOF-2^1^_/2_ framework. This resulted in an order of magnitude with improved conductivity to values of 2.1 × 10^−2^ at 85 °C and 90% RH. This conductivity was the highest equilibrated MOF proton conduction reported, exceeding the sulfuric acid modified MIL-101 framework. The activation energy from the Arrhenius plot was 0.21 eV, indicative for the Grotthuss mechanism of proton conduction. The conductivity in this framework was extremely dependent on the RH and dropped significantly to 2.4 × 10^−5^ S/cm at 20 °C and 50% RH [123].

In another study, a magnesium phosphonate framework was made from Mg(NO_3_)_2_ and octamethylene diamine-N,N,N′,N′-tetrakis-methylenephosphonic acid (H_8_ODTMP) and evaluated for proton conductivity. This magnesium phosphonate framework was possessing 1D channels filled with water molecules running along the c and b axes. The P–OH groups in the framework were found to be pointing inwards to the channel, and thus allowing proton transport to take place, which results in conductivity. The highest proton conductivity for this framework was 1.6 × 10^−3^ S/cm at 19 °C, 100% RH. The proton conductivity in the framework was attributed to the proton transfer in the framework via the Grotthuss hoping mechanism [124]. The influence of metal choice was studied with hybrid metal phosphonates, MFM-Ni and MFM-Co were studied for their proton conductivity properties. At 25 °C and 98% RH, the proton conductivities of Ni and Co hybrids were 4.5 × 10^−4^ and 4.4 × 10^−5^ S/cm, respectively. The differences in conductivities between the isostructural materials arose from differences in the bond strength between the coordination water molecules and the metal cations. The activation energy for proton conduction in the Ni-hybrid was 0.43 eV, this value is at the boundary of the two mechanisms responsible for proton conduction (Grotthuss < 0.4 eV and vehicular > 0.4 eV) [125].

**Figure 10:**
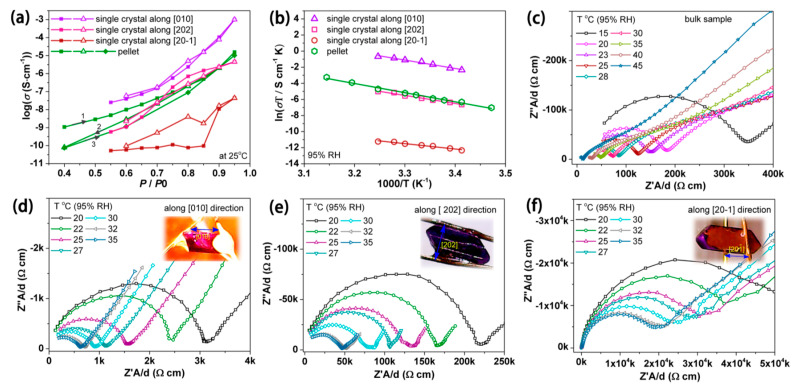
(**a**) Proton conductivity of the single crystals of CoCa·nH_2_O measured along different directions at 25 °C upon increasing (filled) or decreasing (open) humidity. The conductivity of the pellet sample is also shown for comparison; (**b**) plots of log(σT) versus 1000/T for bulk and single crystal samples of CoCa·4H_2_O at 95% relative humidity. The solid lines represent the best fit of the data. Nyquist plots for CoCa·nH_2_O at 95% RH and various temperatures; (**c**) bulk sample; and (**d**–**f**) single crystal sample along the [010], [202], and [20$\bar{1}$] directions, respectively. Reprinted with permission from [126]. Copyright (2016), American Chemical Society.

We conclude this section with the illustration of the capabilities of the metal phosphonate frameworks in thin film applications, such as in sensors or devices Hybrid Co–Ca phosphonates consisting of both Co and Ca and 1,4,7-triazacyclononane-1,4,7-triyl-tris-(methylenephosphonic acid) have been synthesized in the form of powders and as single crystals for their use as proton conducting materials. For the powder samples, the proton conductivity was 1.55 × 10^−5^ S/cm at 25 °C and 95% RH. However, the conductivity was reduced by five orders of magnitude, 0.79 × 10^−10^ S/cm at 25 °C when the RH was 40%. Single-crystals have been grown for these hybrid framework along the (010), (202) and (20$\bar{1}$) directions to determine the conductivity along different planes. The single-crystal grown along the (010) direction displayed the highest conductivity of 1.00 × 10^−3^ S/cm at 25 °C and 95% RH. The lowest conductivity was observed along the (20$\bar{1}$) direction with 4.35 × 10^−8^ S/cm at 25 °C and 95% RH. The higher conductivity along the (010) direction was attributed to a continuous lattice of water molecules and the ‘O’ from the phosphonate present in the channel. The activation energy for the single-crystal hybrids along the (010), (202) and (20$\bar{1}$) directions were 0.90, 0.86 and 0.58 eV, respectively (Figure 10). These values were too large to assign the Grotthuss mechanism for conduction, instead a vehicle mechanism could be in operation. It was highlighted that the presence of ClO_4_^−^ anions, originating from an additive used during synthesis, stayed incorporated in the structure. These anions together with the phosphonate ‘O’ atoms played a key role in connecting the water molecules via a hydrogen bonded network during the conduction process [126].

**Table 3:** Summary of proton conduction in the phosphonate frameworks.

Metal Phosphonate	Conductivity (S cm^−1^)	Activation Energy (eV)	References
ZnCO_3_·2Zn(OH)_2_ + BTP	1.0 × 10^−5^ (at 98% RH, 25 °C)	0.17	[122]
Mg(NO_3_)_2_ + H_8_ODTMP	1.6 × 10^−3^ (at 100% RH, 19 °C)	0.31	[124]
β-PCMOF-2^1/2^	2.1 × 10^−2^ (at 90% RH, 85 °C)	0.21	[123]
CoCa. n H_2_O (pellet) CoCa. n H_2_O (SC)	1.55 × 10^−5^ (pellet) (at 95% RH, 25°C) 1.00 × 10^−3^ (SC) (at 95% RH, 25°C)	0.98 (pellet) 0.90 (SC)	[126]
MFM-500-Ni MFM-500-Co	4.5 × 10^−4^ (at 98% RH, 25 °C) 4.4 × 10^−5^ (at 98% RH, 25 °C)	0.43	[125]
UPG-2	5.7 × 10^−4^ (at 95% RH, 100 °C)	-	[65]

## 4. Summary and Future Outlook

The field of porous metal phosphonates is a growing field of research and is not yet as well established as the carboxylate MOFs, organic–inorganic hybrid silicas, or layered hybrid materials. In this exploratory review, we have highlighted the different classes of porous metal phosphonates coordination networks. Based on layered metal phosphonate systems, we saw the evolution of porous metal phosphonates towards the creation of microporous phosphonate MOFs and porous hybrid inorganic–organic systems (with or without supramolecular templating). The kinetic control, in particular when aiming for mesoporous metal phosphonates, still remains a key challenge in attaining interesting porous architectures. Rapid precipitation due to the heterocondensation/phase separation between metal precursors and phosphonic moieties is one of the remaining concerns. Specifically, when one considers the group IV metals, challenges to harmonize the interactions in a controlled manner are still present when designing porous systems without the use of hazardous HF. Key insights on how the different types of (chelated) metal(IV) precursors react with phosphonate moieties is still a largely unexplored domain. It can thus be envisioned that the challenge to create novel coordination networks of metal phosphonates lies in the reactivity control of the interaction between the metal and phosphonic precursors, in such a way that rapid precipitation is avoided and a controlled build-up of a hybrid porous network is achieved. This problem is further complicated when porosity needs to be induced by the use of SDAs, especially in the case of ionic templates due to their interaction with the phosphonic anion or with the metal ion, influencing reactivity and kinetics. Therefore, the role of templating in these metal phosphonate systems is often not straightforward and more challenging compared to porous metal oxide systems. To date, little studies focused on how ionic and/or polymeric SDAs interact with the phosphonic moieties during the formation of porous metal phosphonate networks. Progress in the successful synthesis of metal phosphonate networks that have been described in this review, proof of the benefit of tailoring the interaction of the phosphonate ligand with the metal precursors (and SDAs) and the need to progress further on this path towards new, more controlled and diverse porous metal phosphonates. This serves to shift the strong kinetically favored condensation reaction, that precipitates as a poorly ordered coordination network, towards the thermodynamically or kinetically controlled build-up of the network. 

The domains for the application of porous metal phosphonates span a wide array of fields. However, we highlighted the main contenders where porous metal phosphonates lend themselves to be the ideal choice. This is often in those applications where multifunctionality (e.g., the combination of structural features with the particular chemistry of the metal and phosphonate unit) provides benefits. Heterogeneous catalysis by hybrid metal phosphonates could benefit from the synergy of combining metal centers with tunable organo-functionalities to optimize the interface for catalyzing reactions. The presence of hydrophilic/acidic porous architecture makes metal phosphonates a good candidate for applications like proton conduction, solid-acid catalysts and metal sorption. In the case of separation/extraction applications, the porous amorphous coordination networks could have potential as solid-phase adsorbents. Mainly in the fields of separation of lanthanides and actinides from highly acidic solutions, wherein the stability of the material under high acidic conditions and radiolytic stability are key issues, they serve as prime candidates for solid-phase extraction (SPE). Efforts in employing crystalline metal phosphonate MOFs as a thin film would be fruitful in order to realize fuel-cell applications, or as sensors, or other applications where ordered channels with specific functional groups are an important feature, such as in fabricated devices. Porous metal phosphonates are thus an important part of the organic–inorganic coordination networks, that promise tremendous potential for material chemists in creating new porous architectures with multifunctional features that can be of benefit to diverse application domains. 

## Supplementary Materials

The following are available online at https://www.mdpi.com/1996-1944/13/23/5366/s1, Table S1: Synthesis protocols for layered metal phosphonates Table S2: Synthesis protocols for phosphonate MOFs; Table S3: Synthesis protocols for templated metal phosphonates.
