# Supplementary material for: Advances and Challenges in the Creation of Porous Metal Phosphonates
Source: Materials (Basel). 2020 Nov 26;13(23):5366. doi: 10.3390/ma13235366 (PMC7734583; doi:10.3390/ma13235366)
Supplement: Supplementary file 1 [file materials-13-05366-s001.pdf]

materials

Supplementary information

Advances and Challenges in the Creation of Porous Metal Phosphonates

Bharadwaj Mysore Ramesha * and Vera Meynen

Laboratory for Adsorption and Catalysis (LADCA), Department of Chemistry, University of Antwerp, Universiteitsplein 1, 2610 Wilrijk, Belgium; vera.meynen@uantwerpen.be
* Correspondence: bharadwaj.mysoreramesha@uantwerpen.be; Tel.: +32-(0)3-265-23-53

Received: 26 October 2020; Accepted: 24 November 2020; Published: 26 November 2020

Table S1. Synthesis Protocol for Porous Metal Phosphonates.

Metal Precursor	Phosphonic Linker	Synthesis Method	Final Morphology	References
$ZrOCl_2$	Mono-alkyl phosphonic acids and their dialkyl ester	Direct precipitation in HF (aqueous media)	Lamellar hexagonal particles (10–100 μm)	[1]
$Th(NO_3)_4$ $ZrOCl_2$	Mono-terphenyl and bis-terphenylene PA	Direct precipitation in HF (dioxane/water)	Lamellar particles	[2]
$ZrOCl_2$	α,ω-bis(phosphonic acid) alkyl or aryl linker	Direct precipitation in HF	Lamellar thin films	[3]
$ZrOCl_2$	Butane DPA, benzene DPA,bis(phosphono methyl) DPA, diphenyl DPA with H_3PO_4	Direct precipitation in HF (DMSO/water)	Lamellar structure with mesoporosity	[4]
Zr (IV) fluoro complexes	3,3′, 5,5′-TMBPDA	Direct precipitation in HF	Lamellar structure with inter-layer microporosity	[5]
$ZnCl_2$	Phenyl-DPA Biphenylyl-DPA	Co-condensation in water	Lamellar structure	[6]
$CuSO_4$ $5H_2O$ & $CuNO_3$ $2.5H_2O$	Phenyl-DPA and Biphenylyl-DPA	Co-condensation in water under reflux or hydrothermal conditions	Lamellar structure	[7]
$CuSO_4$ $5H_2O$ & $ZnCl_2$	Ethylene-DPA and Propylene-DPA	Co-condensation in water	Lamellar structure	[8]
$ZrOCl_2$	Benzene DPA + H_3PO_4	Direct precipitation in HF	Lamellar particles with micro/mesoporosity	[9]
$Zn(NO_3)_2$ & $Cd(NO_3)$	Bis(methylene) phenyl-PA	Co-condensation in water	Lamellar structure	[10]
$ZnCl_2$	Phenyl DPA with H_3PO_4	Hydrothermal in water	Lamellar with slit-shape pores 6.6 nm	[11]
$Mn(ac)_2$ & $Zn(ac)_2$	NMIB-DPA	Hydrothermal in EtOH/water	Lamellar structure with 3D porous framework	[12]
$Co(ac)_2$	Octyl DPA	Co-condensation & Hydrothermal in water (HF)	1D polymeric network and Layered structure	[13]
$SnCl_4$	Phenyl PA	Hydrothermal in HF	Micro and mesoporous	[14]
$Sn(CO_2)_2$	Phenyl DPA	Hydrothermal in water	3D non-porous layered structure	[15]

$SnCl_4$	Phenyl PA<br>Methyl PA	Hydrothermal in HF	Layered spherical globules with micro/mesoporosity	[16]
$SnCl_4$	PPPA	Direct precipitation followed by hydrothermal in HF	Supermicroporous layered particles	[17]
$SnCl_4$<br>$ZrOCl_2$	PPPA<br>PPA	Direct precipitation in HF	Layered micro and mesoporous structure	[18]
$SnCl_4$	Phenyl-PA<br>Biphenyl-PA	Hydrothermal in HF	Layered nano-sized particles with micro/mesoporosity	[19]
$SnCl_4$<br>$ZrOCl_2$	bpyBPAE + Methyl PA	Hydrothermal in DMSO (HF)	Small nanoparticles (<15 nm) with micro/mesoporosity	[20]

Abbreviations: PA = Phosphonic acid; DPA = Diphosphonic acid; 3,3′,5,5′-TMBDPA = Tetramethylbiphenyldiphosphonic acid; NMIB = N-methyliminobis(methylenephosphonic acid); PPPA = 4-(4′-phosphonophenoxy)phenyl phosphonic acid; PPA = Phenylphosphonic acid; and bpyBPAE = Tetraethyl 2,2′-bipyridinediyl-5,5′-bis(phosphonate).

Table S2. Phosphonate—MOFs.

Metal Precursor	Phosphonic Linker	Synthesis Method	Final Morphology	References
Hydrous TiO_2	Methylene DPA	Hydrothermal in HF/water	3D framework with layered structure—Non-porous	[21]
$CoCl_2$ & $Co(ac)_2$<br>$NiCl_2$ & $Ni(ac)_2$	N,N′-PBMDPA	Hydrothermal in water	Hexagonal array of channels with 10 Å pores	[22]
Hydrous TiO_2<br>$AlCl_3$	N,N′-PBMDPA	Hydrothermal in HF/water	3D framework with microporosity	[23]
$Zn(ClO_4)_2$	DHBP	Co-condensation in water & reflux with DMF	3D porous structure with microporosity (10 Å pores)	[24]
$Pb(ac)_2$<br>$ZnCl_2$	EDTP	Hydrothermal in water/EtOH	Open-framework with tunnels & microporous	[25]
$Pb(NO_3)_2$	N,N′-PBMDPA	Hydrothermal in water	3D supramolecular framework	[26]
$ZrOCl_2$	PBMPA	Co-condenastion/Precipitation in water (NH4F or HF)	Layered framework	[27]
$Zn(NO_3)_2$	BDPEt	Co-condensation (THF/water)	3D network with channels of ~4.5A°	[28]
$CuCl_2$	BDPEt<br>BDPMe	Co-condensation (EtOH/water)	Layered pillar structure with VdW interaction	[29]
$CuCO_3{\cdot}Cu(OH)_2$	BDPA<br>Amino triazole	Co-condensation (MeOH/water)	Pillared interlayer architecture with apparent pores	[30]
$Ni(ac)_2$	N,N′-PBMDPA	Hydrothermal in water	3D frameworks with 0.9 nm pores	[31]
$Co(ac)_2$	N,N′-BPBMDPA	Hydrothermal in water	3D honeycomb architecture with pores of 1.8 nm	[32]
$BaBr_2{\cdot}2H_2O$	Octaethyl pyrene-1,3,6,8-tetraphosphonate	Hydrothermal in EtOH/water	3D framework made up by crosslink of 1D chains	[33]
$SnCl_4$	BTBP	Hydrothermal in MeOH	Amorphous microporous (~8.5 Å)	[34]
$CuSO_4$, NiO, $NiSO_4$, & $MnSO_4$	BTTMT<br>TMB-BTTMT	Hydrothermal in water	2D double-layered structure with 1D	[35]

			tunnel with aperture of 3.5 Å × 7.0 Å	
$ZrOCl_2$	BTBP	Direct precipitation in HF	Non-porous honeycomb-like motif	[36]
$ZrOCl_2$	TTBMP	Direct precipitation in HF	Permanent microporosity & channels of (~5–10 Å)	[37]
$Al_2(SO_4)_3$	TMB-TTMT	Hydro(solvo)thermal in EtOH/water	3D framework with hexagonal channels (1.2 nm width)	[38]
$ZrCl_4$	BTBP	Direct precipitation & Hydrothermal (HF)	3D amorphous and semi-crystalline framework with microporosity (10 Å pores)	[39]
$ZrOCl_2$	TTBMP	Direct precipitation in HF	2D layered structure with no porosity	[40]

Abbreviations: DPA = Diphosphonic acid; N,N′-PBMDPA = N,N′-piperazine bis(methylenephosphonic acid); DHBP = 1,4-dihydroxy-2,5-benzenediphosphonate; EDTP = N,N,N′,N′-ethylenediaminetetrakis(methylenephosphonic acid); PBMPA = Piperazine-N,N′-bis(methylenephosphonic acid); BDPEt = 1,4-benzenediphosphonate bis(monoethyl ester); BDPMe = 1,4-benzenediphosphonate bis(monomethyl ester); BDPA = Benzene-1,4-diphosphonic acid; BTTMT = Benzene-1,3,5-triyltris(methylene)triphosphonic acid; TMB-BTTMT = (2,4,6-trimethylbenzene-1,3,5-triyl)tris(methylene)triphosphonic acid; BTBP = 1,3,5-tris(4-phosphonophenyl) benzene; TTBMP = 2,4,6-tris(4-(phosphonomethyl)phenyl)-1,3,5-triazine; and TMB-TTMT = 2,4,6-trimethylbenzene-1,3,5-triyl-tris(methylene)triphosphonic acid.

Table S3. Mesoporous Metal Phosphonates—Templated.

Metal Precursor	Phosphonic Acid	Template	Synthesis Method	Final Morphology	References
$Al(OiPr)_3$	MDPA	ODTMACl	Co-condensation at RT in water	Amorphous, Mesoporous (1.8 nm pores)	[41]
$Al(OsBu)_3$	EDPA	CTAB	Atrane route, Co-precipitation in water	Amorphous, Mesoporous (hexagonal)	[42]
$AlCl_3$	MDPA	Brij-56/58 F68, F127 P123	Co-condensation in EtOH/water followed by EISA	Amorphous Mesoporous (p6m)	[43]
$Al(OiPr)_3$ $AlCl_3$	MDPA EDPA PDPA	CTAB	Co-condensation in water and EtOH/water	Amorphous with Mesoporosity	[44]
$SnCl_4$	PPA	SDS	Hydrothermal	Semi-crystalline, Micro and mesoporous	[45]
$ZrOCl_2$	PPA	-	Hydrothermal in water	Amorphous, Inter-particle mesoporosity	[46]
$Al(OsBu)_3$	H3PMP	CTAB	Atrane route, Co-precipitation in water	Amorphous with mesoporosity	[47]
$Ti(OiPr)_4$	TPPhA	-	Non-hydrolytic condensation in THF	Amorphous with inter-particle porosity	[48]
$V(O)(OiPr)_3$	TPPhA	-	Non-hydrolytic condensation in DMSO	Amorphous with inter-particle porosity	[49]

$Ti(OBu)_4$	HEDP (β-CD)	PS beads	Hydrothermal in EtOH/water	Amorphous, Inter-particle mesoporosity & macroporous	[50]
$Ti(OBu)_4$	HEDP EDTMP	-	Hydrothermal in EtOH/water	Amorphous, plate-like particles & slit shaped inter-particle mesoporosity	[51]
$Ti(OBu)_4$	HEDP	F127 P123	Co-condensation in EtOH followed by EISA	Amorphous, Inter-particle mesoporosity	[52]
$TiCl_4$	EDTMP	Brij-56	Cryogenic condensation in EtOH followed by hydrothermal and EISA	Amorphous, p6mm Mesophase	[53]
$TiCl_4$	HEDP	CTAB	Cryogenic condensation in EtOH followed by hydrothermal treatment	Amorphous, la3d cubic mesophase	[54]
$Ti(OBu)_4$	HEDP EDTMP	-	Hydrothermal in EtOH/water	Amorphous with hierarchical meso/macroporosit y	[55]
$Ti(OBu)_4$	EDTMP DTPMP	-	Hydrothermal in EtOH/water	Crystalline (anatase domains) with phosphonate cap and hierarchical meso/macroporosit y	[56]
$AlCl_3$	BDPA	F127	Co-condensation in EtOH/water	Amorphous, Cubic mesoporous (Im3m)	[57]
$AlCl_3$	DEPT DPAEP DPEP	F127	Co-condensation in EtOH/water	Amorphous, mesoporous thin film	[58]
$Ti(OiPr)_4$	bBzP bPyP	-	Non-hydrolytic sol gel hydrothermal in toluene	Anatase crosslinked bisphosphonates with inter-crystal mesopores	[59]

Abbreviations: MDPA = Methylenediphosphonic acid; EDPA = Ethylenediphosphonic acid; PDPA = Propylenediphosphonic acid; PPA = Phenylposphonic acid; H3PMP = 1-phosphonomethylproline; TPPhA = Tetrakis-1,3,5,7-(4-diethylphosphonatophenyl) adamantane; HEDP = 1-hydroxyethane 1,1-diphosphonic acid; EDTMP = Ethylenediamine tetra(methylene phosphonic acid); DTPMP = Diethylenetriamine penta(methylene phosphonic acid); BDPA = 1,4-phenylene diphosphonic acid; DEPT = 2,5-bis(diethoxyphosphoryl) thiophene; DPAEP = Diethyl (N-diethylphosphonomethylcarbonyl)aminoethyl phosphonate; DPEP = Diethyl 2-(2′-diethylphosphonoethoxy)ethylphosphonate; bBzP = 4,4′-bis(diethylphosphonomethyl)biphenyl; and bByP = tetraethyl 2,2′-bipyridine-5,5′-bisphosphonate.

References

1. Dines, M.B.; Digiacomo, P.M. Derivatized lamellar phosphates and phosphonates of M (IV) ions. *Inorg. Chem.* **1981**, *20*, 92–97, doi:10.1021/ic50215a022.
2. Dines, M.B.; Griffith, P.C. Synthesis and characterization of layered tetravalent metal terphenyl mono- and bis-phosphonates. *Polyhedron* **1983**, *2*, 607–611, doi:10.1016/s0277-5387(00)81519-3.
3. Cao, G.; Hong, H.G.; Mallouk, T.E. Layered metal phosphates and phosphonates: from crystals to monolayers. *Accounts Chem. Res.* **1992**, *25*, 420–427, doi:10.1021/ar00021a007.
4. Alberti, G.; Costantino, U.; Vivani, R.; Zappelli, P. Preparation Of Zirconium Diphosphonate-Phosphites With A Narrow Distribution Of Mesopores. *MRS Proc.* **1991**, *233*, 101–106, doi:10.1557/proc-233-101.
5. Alberti, G.; Casciola, M.; Costantino, U.; Vivani, R. Layered and pillared metal(IV) phosphates and phosphonates. *Adv. Mater.* **1996**, *8*, 291–303, doi:10.1002/adma.19960080405.
6. Poojary, D.M; Zhang, B.; Synthesis and X-ray structures of covalently pillared zinc bis(phosphonates). *Inorg. Chem.* **1996**, *35*, 5254–5263.
7. Poojary, D.M.; Zhang, B.; Bellinghausen, P.; Clearfield, A. Synthesis and X-ray Powder Structures of Two Lamellar Copper Arylenebis(phosphonates). *Inorg. Chem.* **1996**, *35*, 4942–4949, doi:10.1021/ic960319d.
8. Poojary, D.M; Zhang, B.; Pillared layered metal phosphonates. Synthesis and X-ray powder structures of copper and zinc alkylenebis(phosphonates). *J. Am. Chem. Soc.* **1997**, *119*, 12550–12559.
9. Alberti, G.; Marmottini, F.; Vivani, R.; Zappelli, P. Preparation and Characterization of Pillared Zirconium Phosphite-Diphosphonates with Tuneable Inter-Crystal Mesoporosity. *J. Porous Mater.* **1998**, *5*, 221–226, doi:10.1023/a:1009630204407.
10. Penicaud, V.; Massiot, D.; Gelbard, G.; Odobel, F.; Bujoli, B. Preparation of structural analogues of divalent metal monophosphonates, using bis(phosphonic) acids: a new strategy to reduce overcrowding of organic groups in the interlayer space. *J. Mol. Struct.* **1998**, *470*, 31–38, doi:10.1016/s0022-2860(98)00467-0.
11. Zhang, B.; Poojary, D.M.; Clearfield, A. Synthesis and Characterization of Layered Zinc Biphenylylenebis(phosphonate) and Three Mixed-Component Arylenebis(phosphonate)/Phosphates. *Inorg. Chem.* **1998**, *37*, 1844–1852, doi:10.1021/ic9712380.
12. Mao, J.-G.; Wang, Z.; Clearfield, A. Synthesis, Characterization, and Crystal Structures of Two Divalent Metal Diphosphonates with a Layered and a 3D Network Structure. *Inorg. Chem.* **2002**, *41*, 2334–2340, doi:10.1021/ic011202e.
13. Bakhmutova, E.V.; Ouyang, X.; Medvedev, D.G.; Clearfield, A. Cobalt Phosphonates: An Unusual Polymeric Cobalt Phosphonate Containing a Clathrated Phosphonate Anion and a Layered Bisphosphonate. *Inorg. Chem.* **2003**, *42*, 7046–7051, doi:10.1021/ic0301425.
14. Subbiah, A.; Pyle, D.; Rowland, A.; Huang, J.; Narayanan, R.A.; Thiyagarajan, P.; Zoń, J.; Clearfield, A. A Family of Microporous Materials Formed by Sn(IV) Phosphonate Nanoparticles. *J. Am. Chem. Soc.* **2005**, *127*, 10826–10827, doi:10.1021/ja052472p.
15. Subbaiah, A; Bhuvanesh, N; A novel inorganic-organic compound: synthesis and structural characterization of tin(II) phenylbis(phosphonate), $Sn_2(PO_3C_6H_4PO_3)$, J. Solid. State. Chem., 2005, 178, 1321-1325.
16. Huang, J.; Subbiah, A.; Pyle, D.; Rowland, A.; Smith, B.; Clearfield, A. Globular Porous Nanoparticle Tin(IV) Phenylphosphonates and Mixed Methyl Phenylphosphonates. *Chem. Mater.* **2006**, *18*, 5213–5222, doi:10.1021/cm061333j.
17. Gómez-Alcántara, M.D.M.; Cabeza, A.; Olivera-Pastor, P.; Fernández-Moreno, F.; Sobrados, I.; Sanz, J.; Morris, R.E.; Clearfield, A.; Aranda, M.A.G.; Cabeza, A.; et al. Layered microporous tin(iv) bisphosphonates. *Dalton Trans.* **2007**, *23*, 2394–2404, doi:10.1039/b618762e.
18. Cabeza, A.; Gómez-Alcántara, M.D.M.; Olivera-Pastor, P.; Sobrados, I.; Sanz, J.; Xiao, B.; Morris, R.E.; Clearfield, A.; Aranda, M.A.G.; Cabeza, A.; et al. From non-porous crystalline to amorphous microporous metal (IV) bisphosphonates. *Microporous Mesoporous Mater.* **2008**, *114*, 322–336, doi:10.1016/j.micromeso.2008.01.018.
19. Kirumakki, S.; Huang, J.; Subbiah, A.; Yao, J.; Rowland, A.; Smith, B.; Mukherjee, A.; Samarajeewa, S.; Clearfield, A. Tin(IV) phosphonates: porous nanoparticles and pillared materials. *J. Mater. Chem.* **2009**, *19*, 2593–2603, doi:10.1039/b818618a.
20. Perry, H.; Law, J.; Porous zirconium and tin phosphonates incorporating 2,2′-bipyridine as supports for palladium nanoparticles. *Microporous Mesoporous Mat.* **2012**, *149*, 172–180.

21. Serre, C.; Ferey, G.; Hybrid Open Frameworks. 8. Hydrothermal synthesis, crystal structure and thermal behavior of the first three-dimensional titanium (IV) diphosphonate with an open framework structure: $Ti_3O_2(H_2O)_2(O_3P\text{-}(CH_2)\text{-}PO_3)_2{\cdot}(H_2O)_2$, or MIL-22. *Inorg. Chem.* **1999**, *38*, 5370–5373.
22. Groves, J.A.; Miller, S.R.; Warrender, S.J.; Mellot-Draznieks, C.; Lightfoot, P.; Wright, P.A. The first route to large pore metal phosphonates. *Chem. Commun.* **2006**, 3305–3307, doi:10.1039/b605400e.
23. Serre, C.; Groves, J.A.; Synthesis, structure and properties of related microporous N,N′-piperazinebismethylenephosphonates of aluminium and titanium. *Chem. Mater.* **2006**, *18*, 1451–1457.
24. Liang, J.; Shimizu, G.K.H. Crystalline Zinc Diphosphonate Metal−Organic Framework with Three-Dimensional Microporosity. *Inorg. Chem.* **2007**, *46*, 10449–10451, doi:10.1021/ic701628f.
25. Wu, J.; Hou, H.; Highly selective ferric ion sorption and exchange by crystalline metal phosphonates constructured from tetraphosphonic acids. *Inorg. Chem.* **2007**, *46*, 7960–7970.
26. Ma, K.-R.; Zhang, D.-J.; Zhu, Y.-L. Structure and Characterization of a Novel 3D Lead Phosphonate Metal-Organic Framework with Cationic Layer Based on Weak Pb-O(N) Contact. *Aust. J. Chem.* **2010**, *63*, 452–457, doi:10.1071/ch09382.
27. Taddei, M.; Costantino, F.; Vivani, R. Synthesis and Crystal Structure from X-ray Powder Diffraction Data of Two Zirconium Diphosphonates Containing Piperazine Groups. *Inorg. Chem.* **2010**, *49*, 9664–9670, doi:10.1021/ic1014048.
28. Iremonger, S.S.; Liang, J.; Vaidhyanathan, R.; Shimizu, G.K.H. A permanently porous van der Waals solid by using phosphonate monoester linkers in a metal organic framework. *Chem. Commun.* **2011**, *47*, 4430–4432, doi:10.1039/c0cc04779a.
29. 9. Iremonger, S.S.; Liang, J.; Vaidhyanathan, R.; Martens, I.; Shimizu, G.K.H.; Thomas, D.D.; Aghaji, M.Z.; Yeganegi, S.; Woo, T.K. Phosphonate Monoesters as Carboxylate-like Linkers for Metal Organic Frameworks. *J. Am. Chem. Soc.* **2011**, *133*, 20048–20051, doi:10.1021/ja207606u.
30. Vaidhyanathan, R.; Liang, J.; A route to functionalized pores in coordination polymers via mixed phosphonate and amino triazole linkers. *Supramol. Chem.* **2011**, *23*, 278–282.
31. Miller, S.R.; Pearce, G.M.; Wright, P.A.; Bonino, F.; Chavan, S.M.; Bordiga, S.; Margiolaki, I.; Guillou, N.; FéreyG.; Bourrelly, S.; et al. Structural Transformations and Adsorption of Fuel-Related Gases of a Structurally Responsive Nickel Phosphonate Metal−Organic Framework, Ni-STA-12. *J. Am. Chem. Soc.* **2008**, *130*, 15967–15981, doi:10.1021/ja804936z.
32. Wharmby, M.T.; Mowat, J.P.S.; Thompson, S.P.; Wright, P.A. Extending the Pore Size of Crystalline Metal Phosphonates toward the Mesoporous Regime by Isoreticular Synthesis. *J. Am. Chem. Soc.* **2011**, *133*, 1266–1269, doi:10.1021/ja1097995.
33. Taylor, J.M.; Vaidhyanathan, R.; Enhancing the water stability of metal-organic frameworks via phosphonate monoester linkers. *J. Am. Chem. Soc.* **2012**, *134*, 14338–14340.
34. Mah, R.K.; Lui, M.W.; Enhancing order and porosity in a highly robust tin(IV) triphosphonate network. *Inorg. Chem.* **2013**, *52*, 7311–7313.
35. Tang, S. F.; Pan, X. B.; Fabrication of new metal phosphonates from tritopic triphosphonic acid containing methyl groups and auxillary ligands: syntheses, structures, and gas adsorption properties. *Cryst. Eng. Chem.* **2013**, *15*, 1860–1873.
36. Taddei, M.; Costantino, F.; Vivani, R.; Sabatini, S.; Lim, S.-H.; Cohen, S.M. The use of a rigid tritopic phosphonic ligand for the synthesis of a robust honeycomb-like layered zirconium phosphonate framework. *Chem. Commun.* **2014**, *50*, 5737–5740, doi:10.1039/c4cc01253d.
37. Taddei, M.; Costantino, F.; Marmottini, F.; Comotti, A.; Sozzani, P.; Vivani, R. The first route to highly stable crystalline microporous zirconium phosphonate metal–organic frameworks. *Chem. Commun.* **2014**, *50*, 14831–14834, doi:10.1039/c4cc06223j.
38. Tang, S.F.; Cai, J.J.; A highly porous three-dimensional aluminum phosphonate with hexagonal channels: synthesis, structure and adsorption properties. *Dalton Trans.* **2014**, *43*, 5970–5973.
39. Mah, R.K.; Gelfand, B.S.; Reconciling order, stability, and porosity in phosphonate metal organic frameworks via HF-mediated synthesis. *Inorg. Chem. Front.* **2015**, *2*, 273–277.
40. Taddei, M.; Shearan, S.J.; Donnadio, A.; Casciola, M.; Vivani, R.; Costantino, F. Investigating the effect of positional isomerism on the assembly of zirconium phosphonates based on tritopic linkers. *Dalton Trans.* **2020**, *49*, 3662–3666, doi:10.1039/c9dt02463h.
41. Kimura, T.; Synthesis of novel mesoporous aluminum organophosphonate by using a bridged diphosphonic acid, *Chem. Mater.*, **2003**, *15*, 3742–3744.

42. Haskouri, J.E., Guillem, C.; The first pure mesoporous aluminum phosphonates and diphosphonates – new porous hybrid materials. *Eur. J. Inorg. Chem.* **2004**, *9*, 1804–1807.
43. Kimura, T. Oligomeric Surfactant and Triblock Copolymer Syntheses of Aluminum Organophosphonates with Ordered Mesoporous Structures. *Chem. Mater.* **2005**, *17*, 5521–5528, doi:10.1021/cm050919n.
44. Kimura, T. Synthesis of Mesostructured and Mesoporous Aluminum Organophosphonates Prepared by Using Diphosphonic Acids with Alkylene Groups. *Chem. Mater.* **2005**, *17*, 337–344, doi:10.1021/cm0490672.
45. Mal, N.K.; Fujiwara, M.; Yamada, Y.; Matsukata, M. Synthesis of Surfactant-assisted Microporous Layered Tin Phenylphosphonate. *Chem. Lett.* **2003**, *32*, 292–293, doi:10.1246/cl.2003.292.
46. Sarkar, K.; Yokoi, T.; Tatsumi, T.; Bhaumik, A. Mesoporous hybrid zirconium oxophenylphosphate synthesized in absence of any structure directing agent. *Microporous Mesoporous Mater.* **2008**, *110*, 405–412, doi:10.1016/j.micromeso.2007.06.045.
47. Shi, X.; Yang, J.; Mesoporous aluminum organophosphonates functionalized with chiral L-proline groups in the pore. *Eur. J. Inorg. Chem.* **2006**, *10*, 1936–1939.
48. Vasylyev, M.V.; Wachtel, E.J.; Popovitz-Biro, R.; Neumann, R. Titanium Phosphonate Porous Materials Constructed from Dendritic Tetraphosphonates. *Chem. A Eur. J.* **2006**, *12*, 3507–3514, doi:10.1002/chem.200501143.
49. Vasylyev, M.V.; Neumann, R.; Preparation, characterization, and catalytic aerobic oxidation by a vanadium phosphonate mesoporous materials constructed from a dendritic phosphonate. *Chem. Mater.* **2006**, *18*, 2781–2783.
50. Ma, T.Y.; Zhang, X.J.; Ordered microporous titanium phosphonate materials: synthesis, photocatalytic activity, and heavy metal ion adsorption. *J. Phys. Chem. C* **2008**, *112*, 3090–3096.
51. Ma, T.; Zhang, X.-J.; Yuan, Z.-Y. High selectivity for metal ion adsorption: from mesoporous phosphonated titanias to meso-/macroporous titanium phosphonates. *J. Mater. Sci.* **2009**, *44*, 6775–6785, doi:10.1007/s10853-009-3576-7.
52. Ma, T.-Y.; Lin, X.-Z.; Zhang, X.-J.; Yuan, Z.-Y. High surface area titanium phosphonate materials with hierarchical porosity for multi-phase adsorption. *New J. Chem.* **2010**, *34*, 1209–1216, doi:10.1039/b9nj00775j.
53. Ma, T.-Y.; Lin, X.-Z.; Yuan, Z.-Y. Periodic mesoporous titanium phosphonate hybrid materials. *J. Mater. Chem.* **2010**, *20*, 7406–7415, doi:10.1039/c0jm01442g.
54. Ma, T.Y; Lin, X.Z; Cubic mesoporous titanium phosphonates with functionality. *Chem. Eur. J.* **2010**, *16*, 8487–8494.
55. Ma, T.; Lin, X.-Z.; Yuan, Z.-Y. Hierarchical meso-/macroporous phosphated and phosphonated titania nanocomposite materials with high photocatalytic activity. In Proceedings of the Studies in Surface Science and Catalysis; Elsevier BV: Amsterdam, The Netherlands, 2010; Vol. 175, pp. 571–574.
56. Zhang, X.J.; Ma, T.Y.; Titania-phosphonate hybrid porous materials: preparation, photocatalytic activity, and heavy metal ion adsorption. *J. Mat. Chem.* **2008**, *18*, 2003–2010.
57. Kimura, T; Moleular design of bisphosphonates to adjust their reactivity towards metal sources for the surfactant-assisted synthesis of mesoporous films. *Angew. Chem. Int. Ed.* **2017**, *56*, 13459–13463.
58. Wakabayashi, R.; Kimura, T. Further Understanding of the Reactivity Control of Bisphosphonates to a Metal Source for Fabricating Highly Ordered Mesoporous Films. *Chem. A Eur. J.* **2019**, *25*, 5971–5977, doi:10.1002/chem.201900250.
59. Wang, Y.; Alauzun, J.G.; Mutin, P.H. Water-Stable, Nonsiliceous Hybrid Materials with Tunable Porosity and Functionality: Bridged Titania-Bisphosphonates. *Chem. Mater.* **2020**, *32*, 2910–2918, doi:10.1021/acs.chemmater.9b05095.

© 2020 by the authors. Submitted for possible open access publication under the terms and conditions of the Creative Commons Attribution (CC BY) license (http://creativecommons.org/licenses/by/4.0/).
